# Systemic inoculation of *Escherichia coli* causes emergency myelopoiesis in zebrafish larval caudal hematopoietic tissue

**DOI:** 10.1038/srep36853

**Published:** 2016-11-11

**Authors:** Yuelan Hou, Zhen Sheng, Xiaobing Mao, Chenzheng Li, Jingying Chen, Jingjing Zhang, Honghui Huang, Hua Ruan, Lingfei Luo, Li Li

**Affiliations:** 1 Key Laboratory of Freshwater Fish Reproduction and Development, Ministry of Education, Key Laboratory of Aquatic Science of Chongqing, Laboratory of Molecular Developmental Biology, School of Life Sciences, Southwest University, Chongqing, 400715, China; 2 Bioinformatics Department College of Life Science and Biotechnology, Tongji University, 200092, China; 3 Affiliated Hospital of Guangdong Medical University, Zhanjiang, Guangdong, 524001, China

## Abstract

Emergency granulopoiesis occurs in response to severe microbial infection. However, whether and how other blood components, particularly monocytes/macrophages and their progenitors, including hematopoietic stem/progenitor cells (HSPCs), participate in the process and the underlying molecular mechanisms remain unknown. In this study, we challenged zebrafish larvae via direct injection of *Escherichia coli* into the bloodstream, which resulted in systemic inoculation with this microbe. The reaction of hematopoietic cells, including HSPCs, in the caudal hematopoietic tissue was carefully analysed. Both macrophages and neutrophils clearly expanded following the challenge. Thus, emergency myelopoiesis, including monopoiesis and granulopoiesis, occurred following systemic bacterial infection. The HSPC reaction was dependent on the bacterial burden, manifesting as a slight increase under low burden, but an obvious reduction following the administration of an excessive volume of bacteria. Pu.1 was important for the effective elimination of the microbes to prevent excessive HSPC apoptosis in response to stress. Moreover, Pu.1 played different roles in steady and emergency monopoiesis. Although Pu.1 was essential for normal macrophage development, it played suppressive roles in emergency monopoiesis. Overall, our study established a systemic bacterial infection model that led to emergency myelopoiesis, thereby improving our understanding of the function of Pu.1 in this scenario.

When vertebrates are infected by pathogens such as bacteria, the immune cells, particularly mononuclear (monocytes/macrophages) and polymorphonuclear (granulocytes) myeloid phagocytes, respond immediately^1^. A slight infection such as a localised bacterial challenge induces the recruitment of myeloid phagocytes, particularly granulocytes, from the bloodstream, but exerts a limited influence on their exhaustion and expansion^2^,^3^. However, severe infection, including systemic bacterial inoculation, usually results in a bacteraemia-like syndrome. Granulocytes are intensively involved in this process, resulting in their significant exhaustion and expansion, which is called emergency granulopoiesis^2^,^3^. Emergency granulopoiesis is achieved via the activation of granulocyte progenitors, including hematopoietic stem and progenitor cells (HSPCs)^2^,^3^,^4^.

As the ancestral cells of all blood components, HSPCs are a heterogeneous population, and only a limited portion comprises *bona fide* hematopoietic stem cells (HSCs), which reside quiescently in the bone marrow under steady conditions^4^,^5^,^6^,^7^,^8^,^9^. Although these physiologically dormant HSCs quickly enter the cell cycle upon challenge^4^,^5^,^6^,^7^,^8^,^9^, how they react to pathogens remains unclear, primarily because of the difficulty in isolating pure HSCs^3^,^4^. However, the response of HSPCs and their subsequent developmental potential under demand-derived emergency conditions, such as when encountering pathogens or when stimulated by cytokines, has been the focus of research recently^1^,^4^,^6^. Different pathogens induce different HSPC reactions^6^. Additionally, the route and severity of the infection lead to different HSPC outcomes^1^,^4^,^6^. Moreover, HSPCs are prone to granulocyte production by sacrificing lymphoid cells in infection-induced emergency granulopoiesis^1^,^2^. Although the reaction of bone marrow-derived HSPCs has been examined^1^,^4^,^6^, how HSPCs respond during the embryonic stages has rarely been addressed.

Zebrafish (*Danio rerio*) provides an ideal system for studying bacterial infection-stimulated immune response, particularly the interaction of phagocytes with microbes, owing to its optical transparency and the exclusive involvement of myeloid phagocytes in early embryos^10^,^11^,^12^,^13^. Using this model, the behaviours of phagocytes, including their response, mobility, movement, interaction, and engulfment, have been reported^10^,^11^,^12^,^13^. Recently, a study investigated emergency granulopoiesis through local infection of larvae in the hindbrain^10^. However, to our knowledge, myelopoiesis in systemically infected larvae and the role of HSPCs in this phenomenon have not been elucidated.

HSPCs emerge in zebrafish from approximately 36 hours post-fertilization (hpf) via endothelial-to-hematopoietic transition^14^,^15^. From 2 days post-fertilization (dpf), these HSPCs migrate to the caudal hematopoietic tissue (CHT), an organ transiently supporting hematopoiesis and the functional homolog of the fetal liver and placenta^16^,^17^,^18^,^19^. CHT refers to the lumen between the caudal artery (CA) and the definitive caudal vein (dCV). Structurally, it is a complex vascular network composed of a fibroblastic reticular-cell matrix, loose mesenchyme, and expanded blood progenitors^16^,^17^,^18^,^19^. After a transient stay, these HSPCs move to the kidney, which is the functional equipment of the bone marrow^17^,^19^,^20^, from 3–4 dpf on, and eventually, give rise to all blood components. Some of the HSPCs migrate to the thymus to generate T lymphoid cells^21^. Functional T lymphoid cells are detected after 6 dpf, and the adaptive immunity functions until the juvenile period^22^.

Taking advantage of the well-addressed process of hematopoiesis^20^, bacterial inoculation of early embryos provides an effective assay for investigating infection-induced emergency hematopoiesis, in addition to elucidating the behaviour of myeloid phagocytes. Consequently, an improved understanding of the cellular and molecular mechanisms responsible for emergency hematopoiesis can be achieved. In this study, we established a systemic infection model by injecting the non-pathogenic bacteria, *Escherichia coli*, into the bloodstream of zebrafish embryos. CHT^16^,^17^,^18^,^19^ was examined to elucidate the influence of the bacteria on various phagocytes and their progenitors, including HSPCs.

## Results

### Both macrophages and neutrophils in the CHT are involved in bacterial phagocytosis upon systemic infection

**Figure 1:**
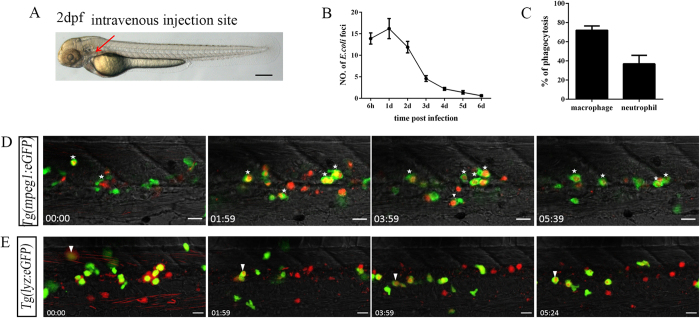
Bacterial (*E. coli*) phagocytosis by both macrophages and neutrophils following intravenous injection. (**A**), The intravenous injection site (red arrow) of *E. coli* (5–10 × 10^3^ cfu) in 2 dpf embryo. Scale bars, 200 μm. (**B**), The numbers of Dsred^+^ *E. coli* foci in the CHT regions at different time points after injection (13.90 ± 1.31; 16.20 ± 2.35; 11.90 ± 1.33; 4.60 ± 0.65; 2.20 ± 0.33; 1.40 ± 0.40; 0.60 ± 0.22 at each time points; 10 embryos were counted in each group). (**C**), The percentage of macrophages (72.06 ± 4.44, N = 8) and neutrophils (36.93 ± 8.89, N = 8) involved in bacterial phagocytosis at 6 hpi in the CHT. (**D,E**), Time-lapse imaging of an infected *Tg(mpeg1:eGFP)* (**D**) or *Tg(lyz:eGFP)* (E) CHT from 0.5 hpi to 6 hpi. The white stars in (**D**) denote mpeg1-GFP^+^ macrophages that engulfed large amounts of Dsred^+^ *E. coli*. The white arrowheads in (**E**) indicate the initial aggregation of Dsred^+^ *E. coli* on the surface of lyz-GFP^+^ neutrophils, which were quickly phagocytosed. The red foci in (**D**,**E**) represent the phagocytosed bacteria. Scale bars, 20 μm. See also Video S1 and S2.

To investigate the hematopoietic reaction following systemic inoculation of larvae with microbes, 2 dpf larvae were challenged by intravenous injection of Dsred-labelled *E. coli*^13^,^23^. A site close to the ear, where vessels are enriched (Fig. 1A), was chosen as the injection site to facilitate rapid inoculation of the microbes into the blood circulation. When the microbes were administered, tremendous amounts of circulating bacteria were identified by intensive red fluorescent signals moving through the vessels (see supplementary Video S1 and S2). Gradually, the circulating microbes disappeared; instead, large clusters of Dsred^+^ bacterial foci were detected^13^,^23^ (Fig. 1B; see supplementary Video S1 and S2). In parallel with the disappearance of moving bacteria in the circulation, the number of large foci increased to approximately 16 in the CHT^16^,^19^,^24^ at 1 day post-injection (dpi). However, this number decreased thereafter, and the bacteria almost completely disappeared by 6 dpi (Fig. 1B). The alteration of foci reflected bacterial phagocytosis and digestion by the myeloid phagocytes, which are the only immunocytes that function at these stages^13^,^23^. To monitor the behaviour of both myeloid phagocytes, *Tg(mpeg1:eGFP)*^25^ and *Tg(lyz:eGFP)*^*nz117*^ 26, which specifically label macrophages and neutrophils, respectively, were exploited. The mpeg1-GFP^+^ macrophages reacted immediately to interact with and engulf the microbes^13^,^23^ (Fig. 1D, white stars). At approximately 30 minutes post-injection, the macrophages had engulfed numerous microbes, resulting in the formation of large red foci in the CHT (Fig. 1D; see supplementary Video S1; white stars). Over time, the number of active macrophages increased markedly, and approximately 72% of them were observed to actively engulf microbes at 6 hours post-injection (hpi) (Fig. 1C), which was consistent with the numerous large *E. coli* foci observed in the CHT (Fig. 1B). Gradually, macrophages with a huge microbe burden underwent cell death, manifested by weakened and even lost GFP signals, and these scarified macrophages were quickly engulfed by their surrounding macrophages (see supplementary Video S1, white arrowheads). The lyz-GFP^+^ neutrophils also phagocytosed bacteria following treatment with *Tg(lyz:eGFP)*^*nz117*^ 26. Interestingly, the phagocytic behaviour of neutrophils was distinct from that of macrophages. Neutrophils first aggregated the bacteria on their surface, resulting in their encircling by more extensive Dsred^+^ signals (Fig. 1E; see supplementary Video S2, white arrowheads), followed by engulfment of the bacteria^13^ (see supplementary Video S2, white arrowheads). However, the reaction sensitivity and phagocytic ability of neutrophils were less efficient than those of macrophages^27^. Only approximately 37% of the total lyz-GFP^+^ neutrophils in the CHT performed phagocytosis, which was half the rate determined for macrophages at similar time points (Fig. 1C). Therefore, both macrophages and neutrophils were involved in phagocytosis when encountering bacteria in the CHT.

### Inoculation of microbes into the bloodstream leads to emergency granulopoiesis

**Figure 2:**
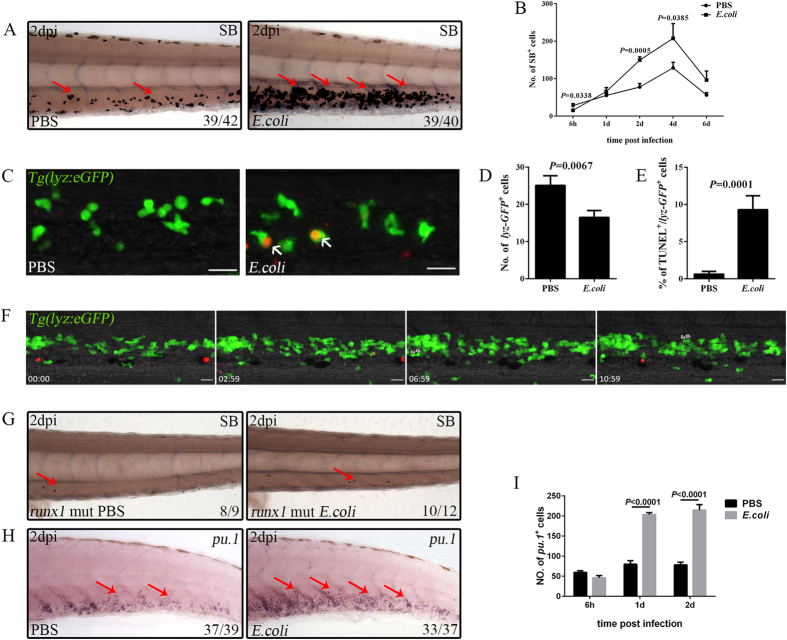
Emergency granulopoiesis occurs following intravenous *E. coli* (5–10 × 10^3^ cfu) infection. (**A**), The drastic expansion of SB^+^ neutrophils (red arrows) in the CHT of an infected embryo when compared with that treated with PBS at 2 dpi (4 dpf). (**B**), Calculation of the data obtained for SB^+^ neutrophils at different time points after infection (15.60 ± 2.80 vs 28.20 ± 5.27; 65.71 ± 10.60 vs 55.20 ± 4.76; 149.50 ± 8.70 vs 77.60 ± 9.45; 207.60 ± 39.15 vs 128.80 ± 15.00; 96.40 ± 23.80 vs 56.40 ± 6.33 in *E. coli* vs PBS group at each time point. N = 8 in each group). (**C,D**), Fluorescence images (**C**) and calculation (**D**) of lyz-GFP^+^ neutrophils in PBS (25.11 ± 2.58; N = 9) or *E. coli* (16.50 ± 1.84; N = 10) treated larval CHT at 6 hpi. The red signals indicate the bacteria phagocytosed by lyz-GFP^+^ neutrophils (white arrows). (**E**), The percentage of lyz-GFP^+^ neutrophils that are co-stained with TUNEL at 6 hpi (9.31 ± 1.87 vs 0.63 ± 0.37 in *E. coli* vs PBS group. N = 10 in each group). (**F**), Time-lapse imaging of an infected *Tg(lyz:eGFP)* CHT from 1 dpi to 1.5 dpi. Obvious generation, expansion and maturation of lyz-GFP^+^ neutrophils are observed. Scale bars, 20 μm. See also Video S4. 4a, 4b and 8a, 8b in (**F**) showing the dividing lyz-GFP^+^ cells. (**G**), SB^+^ signals (red arrows) in the *runx1* mutant treated by either PBS or *E. coli* at 2 dpi (4 dpf). (**H**), WISH of *pu.1* (red arrows) in the CHT of an embryo challenged with PBS or *E. coli* at 2 dpi (4 dpf). (**I**), The number of the *pu.1*^+^ myeloid progenitors at different time points after challenge (45.88 ± 5.87 vs 59.63 ± 4.29; 203.38 ± 5.02 vs 79.88 ± 8.70; 214.50 ± 13.98 vs 78.16 ± 6.89 in *E. coli* vs PBS group at each time point. N = 8 in each group). Scale bars, 20 μm.

Emergency granulopoiesis has been detected in larvae infected through the hindbrain^10^. Whether a similar phenomenon was recapitulated in the CHT during systemic infection was investigated. We first examined several neutrophil markers, including *cebp1, lyz, mpx,* and Sudan Black (SB)^28^, at 2 dpi. All examined neutrophil markers displayed a drastic increase in the treated larvae compared with the controls (Fig. 2A; see supplemental Figure S1A–C). Moreover, the degree of neutrophil expansion depended on the bacterial burden. More significant expansion of SB^+^ cells was observed with 5–10 × 10^3^ colony-forming units (cfu) than with 5–10 × 10^2^ cfu *E. coli* (see supplemental Figure S1D). However, when the *E. coli* volume reached 5–10 × 10^4^ cfu, the larvae showed remarkable mortality (see supplemental Figure S1E), and approximately half of the surviving larvae presented obvious morphological abnormalities, exemplified by pericardium oedema (see supplemental Figure S1F). A larger volume of microbes caused a stronger neutrophil reaction, but an excessive burden led to fish abnormality and lethality. Therefore, 5–10 × 10^3^ cfu *E. coli* was chosen as the dosage in all further experiments because it caused a remarkable immune response with few morphological defects and low lethality, thus permitting continuous investigation of the challenge-induced emergency hematopoietic reaction.

Next, the number of SB^+^ neutrophils was calculated. A transient reduction of SB^+^ neutrophils was detected at 6 hpi (Fig. 2B), suggesting their early exhaustion. To confirm this phenomenon, the challenged *Tg(lyz:eGFP)*^*nz117*^ 26 were examined carefully. The lyz-GFP^+^ population showed a similar reduction as that of SB^+^ neutrophils (Fig. 2D). Further investigation revealed obvious phagocytosis of the Dsred^+^ microbes by the lyz-GFP^+^ neutrophils (Fig. 2C, white arrows) and a corresponding increase in the portion of lyz-GFP^+^/terminal deoxynucleotidyl transferase dUTP nick-end labelling (TUNEL)^+^ cells in the microbe-treated larvae when compared with their controls (Fig. 2E). These results suggested that the early exhaustion of neutrophils was caused by their increased apoptosis while fighting the bacteria. Subsequently, the SB^+^ neutrophil population began to expand beginning at 1 dpi, reaching their maximal level at 2–4 dpi, and then returning to physiological baseline numbers from 6 dpi on (Fig. 2B). The expansion of neutrophils indicated that emergency granulopoiesis occurred in response to systemic infection. CHT is a transient organ for definitive hematopoiesis^16^,^17^,^18^,^19^, and therefore, the origin of expanded neutrophils in this region was explored. Time-lapse images were acquired from 1 dpi for the treated *Tg(lyz:eGFP)*^*nz117*^ 26, and the data revealed the generation of nascent lyz-GFP^+^ neutrophils in the niche adjacent to the CA (Fig. 2F; see supplementary Video S4), where more immature progenitors resided^24^. The lyz-GFP^+^ neutrophils showed weak signals initially that gradually increased in strength, indicating differentiation of the neutrophils after challenge. Meanwhile, the lyz-GFP^+^ neutrophils divided more frequently in *E. coli*-treated larvae. An average of 8.00 ± 0.41 divisions were observed in four imaged larvae, which is approximately four-times higher than that in the control groups (only 2.33 ± 0.33 divisions were observed in three control larvae during the imaging time window) (Fig. 2F; see supplementary Video S3 and S4). As a result, the lyz-GFP^+^ neutrophil numbers increased. The *in situ* expansion of neutrophils in the CHT predicted a definitive hematopoietic origin^10^. To test our hypothesis, we utilised a *runx1*^*w84x*^ mutant, in which definitive hematopoiesis is abolished^24^. The results revealed no detectable expansion of SB^+^ neutrophils in challenged *runx1*^*w84x*^ larvae compared with the control (Fig. 2G). This finding suggested that the expanded neutrophils were largely generated from *runx1*-regulated definitive hematopoiesis^24^, in agreement with a previous study^10^. The enhanced output of neutrophils supported the activation and expansion of their progenitors. This hypothesis was verified by the significantly enhanced output of both *pu.1*^+^ and *cebpα*^+^ myeloid progenitors at 2 dpi (Fig. 2H; see supplemental Figure S2A). Further calculations demonstrated that the increase in myeloid progenitors was initiated as early as 1 dpi (Fig. 2I), a time point prior to the tremendous output of neutrophils (Fig. 2B). However, the myeloid progenitors did not display a reduction at 6 hpi (Fig. 2I), suggesting limited exhaustion of myeloid progenitors in the initial fight against the bacteria. The expansion of myeloid progenitors predicted their higher proliferation upon challenge. To support this hypothesis, *Tg(coro1a:eGFP)*^29^, which marks both myeloid phagocytes and their progenitors, was evaluated by anti-phospho-histone H3 (pH3) antibody staining^30^. A remarkable increase in pH3^+^/coro1a-GFP^+^ cells was detected in the microbe-treated larvae, when compared with the control, at 1 dpi (see supplemental Figure S2 B,C). Together, these results suggested that the myeloid progenitors were activated, and emergency granulopoiesis occurred in the larvae following systemic infection.

### Distinct reactions of the HSPC compartment in response to different bacterial burdens

**Figure 3:**
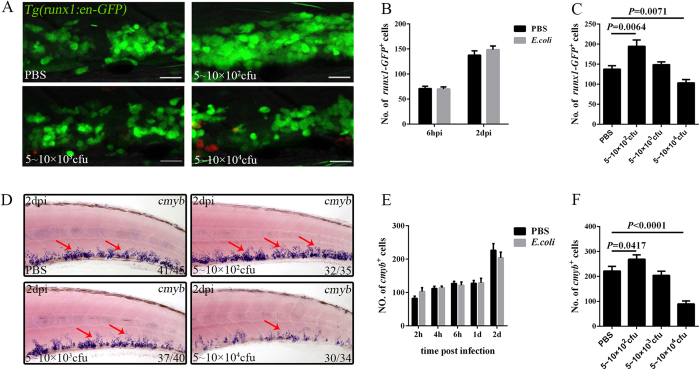
Reaction of HSPCs to different *E. coli* burdens after intravenous injection. (**A**), Fluorescence images showing the runx1-GFP^+^ cells in *Tg(runx1:en-GFP)* larvae treated with different volumes of *E. coli* at 2 dpi (4 dpf). Scale bars, 20 μm. (**B**), The number of runx1-GFP^+^ cells in larvae treated with 5–10 × 10^3^ cfu *E. coli* or PBS. (70.31 ± 4.18 vs 71.14 ± 4.42; 148.8 ± 7.14 vs 137.6 ± 8.65 in *E. coli* vs PBS group at each time points. N ≥ 7 in each group). (**C**), Calculation of the runx1-GFP^+^ cells in (**A**) (137.6 ± 8.65; 194.6 ± 15.63; 148.8 ± 7.14; 103.4 ± 8.38 in each group. N ≥ 8 in each group). (**D**), WISH of *cmyb* (red arrows) in the larval CHT treated with different volumes of *E. coli* at 2 dpi (4 dpf). (**E**), The data obtained for *cmyb*^+^ cells at different time points after treatment with 5–10 × 10^3^ cfu *E. coli* or PBS (102.80 ± 11.67 vs 82.40 ± 6.59; 114.00 ± 4.97 vs 112.00 ± 6.60; 121.60 ± 9.70 vs 127.00 ± 6.69; 129.00 ± 13.71 vs 127.60 ± 8.20; 203.80 ± 17.06 vs 226.40 ± 20.07 in *E. coli* vs PBS group at each time point. N = 8 in each group). (**F**), Calculation of the *cmyb*^+^ cells in (**D**) (221.60 ± 18.56; 268.70 ± 17.77; 203.80 ± 17.06; 89.30 ± 12.54 in each group. N = 10 in each group).

Because no specific HSC-labelling method is available in zebrafish, we mainly utilised *Tg(runx1:en-GFP)*, in which runx1-GFP largely marks the HSPC compartment^31^, to dissect their reactions in our assay. To our surprise, runx1-GFP^+^ cells did not show an obvious increase in the CHT after challenge by 5–10 × 10^3^ cfu *E. coli* (Fig. 3A–C), even when the myeloid progenitors were drastically expanded (Fig. 2H; see supplemental Figure S2A), suggesting distinct reactions of HSPCs and myeloid progenitors upon infection. To confirm this result, we employed another HSPC compartment marker, *cmyb*, using both whole-mount *in situ* hybridization (WISH) and its reporter line^32^. The results revealed a similar number of *cmyb*^+^ cells in both control and challenged larvae (Fig. 3D–F; see supplemental Figure S3A–C), suggesting that HSPCs maintained their homeostasis during this process. The reaction of HSPCs upon challenge was distinct from that observed previously^10^,^33^,^34^, and the underlying reasons were explored by first considering the bacterial burden^6^. Different volumes of *E. coli* were administered. When the volume was 5–10 × 10^2^ cfu, the runx1-GFP^+^ HSPCs showed a notable expansion (Fig. 3A,C). Surprisingly, when the burden increased, the number of HSPCs decreased, and 5–10 × 10^4^ cfu *E. coli* led to a significant reduction in runx1-GFP^+^ cells in surviving larvae with a normal appearance at 2 dpi (Fig. 3A,C). Similar alterations were also observed in *cmyb*^+^ cells (Fig. 3D,F). Together, these results indicated that the HSPC reaction was dependent on the bacterial burden. Next, the possible mechanisms underlying the HSPC reactions were examined. Several inflammatory cytokines have been suggested to be critical for the activation of HSPC proliferation^5^,^35^,^36^,^37^; however, their overproduction causes HSPC apoptosis^5^,^6^,^38^,^39^. Therefore, the expression levels of various cytokines were measured in response to different bacterial burdens by quantitative real-time polymerase chain reaction (qPCR). The results revealed an obvious increase in the expansion of these factors, including *tnfα, ifng1–2,* and *il1b* (see supplemental Figure S3D), following a more severe challenge at 2 dpi. This result was consistent with the reaction of HSPCs, further supporting that an optimal level of inflammatory factors is essential for their homoeostasis and that overdose might lead to their exhaustion^5^,^6^,^37^,^38^,^39^.

### Emergency monopoiesis occurs during systemic infection

**Figure 4:**
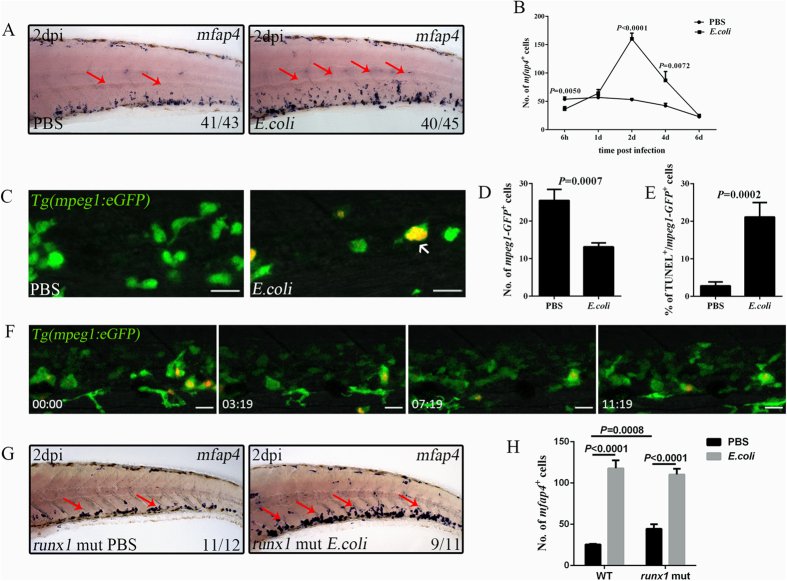
Emergency monopoiesis occurs upon intravenous *E. coli* (5–10 × 10^3^ cfu) infection. (**A**), WISH of *mfap4* (red arrows) in the CHT of a larva treated with PBS or *E. coli* at 2 dpi (4 dpf). (**B**), The data obtained for *mfap4*^+^ macrophages at different time points after challenge (36.13 ± 4.52 vs 53.88 ± 3.90; 64.25 ± 6.58 vs 57.00 ± 3.29; 160.44 ± 9.92 vs 53.20 ± 1.90; 87.00 ± 15.67 vs 42.44 ± 4.21; 24.71 ± 2.14 vs 23.00 ± 2.30 in *E. coli* vs PBS group at each time point. N ≥ 8 in each group). (**C,D**), Fluorescence images (**C**) and calculation (**D**) of mpeg1-GFP^+^ macrophages in PBS (25.50 ± 2.94; N = 8) or *E. coli* (13.13 ± 1.09; N = 8) treated larval CHT at 6 hpi. The red signals indicate the bacteria phagocytosed by mpeg1-GFP^+^ macrophages (white arrows). (**E**), The percentage of mpeg1-GFP^+^ macrophages that co-stained with TUNEL at 6 hpi (21.13 ± 3.86 vs 2.82 ± 1.05 in *E. coli* vs PBS group. N ≥ 10 in each group). (**F**), Time-lapse imaging of an infected *Tg(mpeg1:eGFP)* CHT from 1 dpi to 1.5 dpi (see also Video S5). (G-H), WISH (**G**) and calculation data (**H**) for *mfap4*^+^ macrophages (red arrows) in the *runx1* mutant treated with either PBS or *E. coli* at 2 dpi (4 dpf) (WT: 117.9 ± 9.63 vs 25.38 ± 0.96 *E. coli* vs PBS; *runx1* mut: 110.3 ± 6.88 vs 44.40 ± 5.72 *E. coli* vs PBS; N ≥ 5 in each group). Scale bars, 20 μm.

Similar emergency granulopoiesis, but with a different HSPC reaction, was observed in our assay compared with the results of a previous study^10^. Whether this difference was caused by the distinct infection methods or variations in the microbes used was explored further. When the microbes, whether they were *Salmonella typhimurium* or *Dsred-labelled E. coli*^13^,^23^, were injected into the 2 dpf larval hindbrain or the blood circulation, emergency granulopoiesis occurred, as evidenced by clear expansion of SB^+^ neutrophils (see supplemental Figure S4A). Thus, emergency granulopoiesis occurred following either brain or systemic infection of the larvae. When the *mpeg1*^+^ macrophages were examined, no expansion of *mpeg1*^+^ macrophages was observed in the CHT when the larvae were challenged via the hindbrain by both microbes (see supplemental Figure S4B)^10^, indicating that brain infection did not lead to emergency monopoiesis in the CHT. However, intravenous injection of microbes caused a tremendous expansion of *mpeg1*^+^ macrophages in the CHT (see supplemental Figure S4B). Thus, emergency monopoiesis, in addition to granulopoiesis, likely occurred when the microbes were systemically inoculated. This conclusion was further confirmed by the drastic expansion of another macrophage marker, *mfap4*, at 2 dpi (Fig. 4A,B). The reasons for the different reactions of the macrophages in response to different infection methods were investigated further. Although an obvious restriction of the bacteria was detected in the injured brain, hindbrain administration led to limited circulating microbes in the CHT, in contrast to the drastic increase in circulating microbes after intravenous injection (see supplemental Figure S4C,D). Therefore, the tremendous number of bacteria in the circulation and the intensive involvement of macrophages in bacterial phagocytosis (Fig. 1D) probably provided cues that led to emergency monopoiesis. To carefully dissect the process of emergency monopoiesis, *mfap4*^+^ and mpeg1-GFP^+^ cells were quantified. Similar to the fluctuation of neutrophils, *mfap4*^+^ and mpeg1-GFP^+^ macrophages^25^ displayed an initial exhaustion (Fig. 4B,D), which was in agreement to their intensive involvement in phagocytosis and resultant increased apoptosis (Fig. 4C,E). Subsequently, a significant expansion of *mfap4*^+^ macrophages followed (Fig. 4B). However, their recovery to baseline was faster than that of SB^+^ neutrophils. At 4–6 dpi, the macrophage numbers had already declined to the levels of the control (Fig. 4B), approximately 2 days earlier than the neutrophils (Fig. 2B). Other lineage markers presented no obvious alterations (see supplemental Figure S5). Overall, systemic infection of microbes caused both emergency monopoiesis and granulopoiesis, which are collectively referred to as emergency myelopoiesis.

### Emergency monopoiesis is achieved through the expansion of primitive myeloid cells

The definitive hematopoietic origin of the emergency granulopoiesis suggested a similar origin for emergency monopoiesis. To verify this hypothesis, time-lapse imaging was performed in the infected *Tg(mpeg1:eGFP)* larval CHT. Expansion of mpeg1-GFP^low^ macrophages was detected, and their numbers increased upon infection (Fig. 4F; see supplementary Video S5). These mpeg1-GFP^low^ macrophages should have been nascent. However, they highly expressed GFP signals following engulfment of bacteria (Fig. 4F; see supplementary Video S5). The mpeg1-GFP^low^ macrophages suggested the definitive hematopoietic origin of emergency monopoiesis. However, when the *mfap4*^+^ macrophages were examined in the *runx1*^*w84x *24^, they presented a surprisingly remarkable expansion following challenge, and the numbers of expanded macrophages were similar to those in the siblings (Fig. 4G,H). This macrophage phenomenon is the converse of that observed for neutrophils in similar mutant larvae, suggesting that the emergency monopoiesis was largely independent of *runx1*-mediated hematopoiesis and that these cells were probably generated from primitive myeloid cells. Thus, emergency granulopoiesis and monopoiesis at this stage had different origins.

### The immune response and hematopoiesis-related factors are transcriptionally influenced after challenge

**Figure 5:**
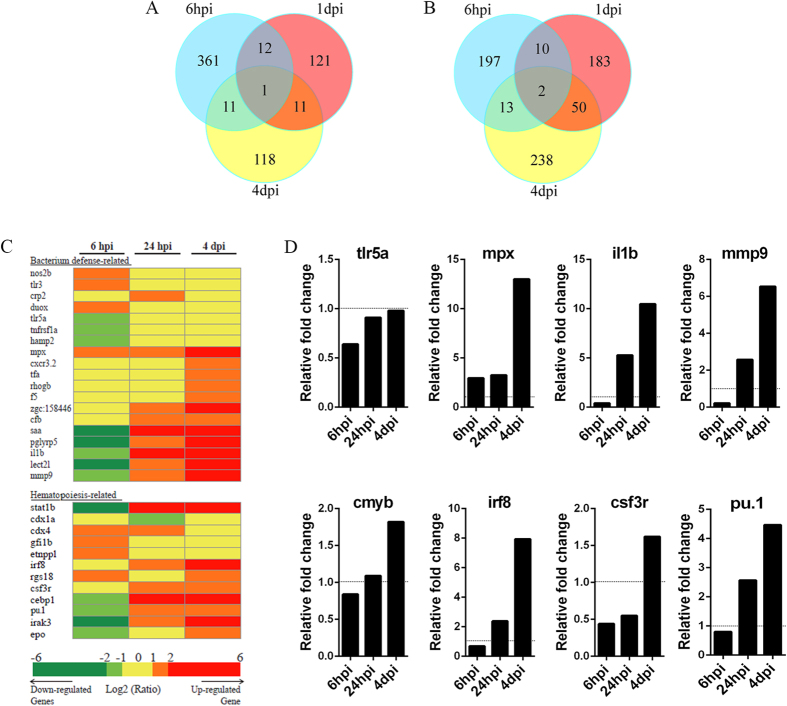
Comparison of bacterial defense-related and hematopoiesis-related response factors at different time points after intravenous injection of *E. coli* (5–10 × 10^3^ cfu). (**A,B**), Venn diagrams showing the overlap and differences between successive time points after *E. coli* infection in the numbers of downregulated (**A**) or upregulated (**B**) genes. (**C**), Gene expression profiles of *E. coli*-infected embryos at different time points are depicted in a heat map. Bacterial defense-related and hematopoiesis-related genes in the heat map are ordered in functional groups. All genes included in the heat map are represented by a minimum of two probes that showed significant up- or down-regulation (significance cut-offs for the ratios of infected versus control groups were set at 2-fold). Up- and down-regulation are indicated by increasingly bright shades of red and green, respectively. (**D**), qPCR analysis indicating the similar alterations of the expression levels of *tlr5a, mpx, il1b, mmp9, irf8, csf3r, pu.1* and *cmyb* in (**C**).

The molecular mechanisms underlying emergency myelopoiesis were further explored. To this end, deep-sequence analysis was performed using samples collected at successive time points after treatment. Three typical representative time points—6 hpi, 1 dpi, and 4 dpi—were chosen on the basis of both the alteration of myeloid phagocytes and the level of bacterial clearance. The results indicated that large amounts of factors changed during the different stages after challenge (Fig. 5A,B; Table S1 and S2). Altered factors functioning in bacterial defense and hematopoiesis were examined further. The heat-map results revealed that dozens of bacterial defense-related and hematopoiesis-related genes were transcriptionally modified throughout the process (Fig. 5C and Table S2). These genes could be classified mainly into two types. The first type showed a typical increase at 6 dpi but a quick reduction thereafter. Nos2b and Duox—two important factors that are closely involved in the formation of H_2_O_2_ and NO—were the representative examples (Fig. 5C), suggesting essential roles for small molecules as initial emergency signals^10^,^34^,^40^. Another type manifested an initial reduction followed by an obvious increase at later stages. This group of factors accounted for a large portion of the total members, and was typified by pro-inflammatory cytokines such as *il1b* and *mmp9*, as well as most hematopoiesis-related genes (Fig. 5C). qPCR was performed to validate the deep-sequence analysis data. The results revealed similar alterations in the expression of key factors, including *tlr5a, mpx, il1b, mm9, irf8, csf3r*, and *pu.1,* to that in the deep-sequence analysis results (Fig. 5D). However, the HSPC marker *cmyb* exhibited a slight upregulation (<2-fold) (Fig. 5D), which was consistent with its behaviour in the deep-sequence analysis, and this finding further supported the results suggesting limited alterations of HSPC numbers in our assay.

### Different roles of Pu.1 in steady and emergency monopoiesis

**Figure 6:**
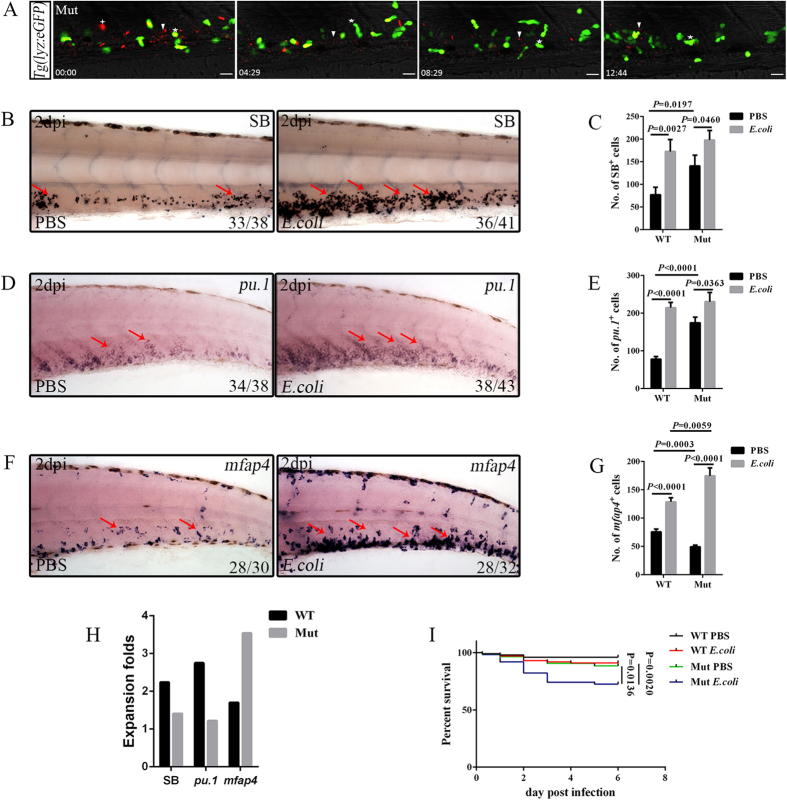
Pu.1 suppresses emergency monopoiesis after intravenous infection of *E. coli* (5–10 × 10^3^ cfu). (**A**), Time-lapse imaging of an infected 2 dpf *pu.1*^*G242D/G242D*^*/Tg(lyz:eGFP)* CHT from 6 hpi to 19 hpi. The white stars indicate that lyz-GFP^+^ neutrophils phagocytosed bacteria to form small foci. The white arrowheads represent free bacteria. The white cross star denotes the large *E. coli* foci. Scale bars, 20 μm (see also Video S7). (**B,C**), SB staining (B) and calculation of SB^+^ neutrophils (**C**) in the CHT of treated WT (172.90 ± 26.23 vs 77.10 ± 16.47 in *E. coli* vs PBS group. N = 8 in each group) or *pu.1*^*G242D/G242D*^ larvae (198.00 ± 21.13 vs 140.80 ± 23.92 in *E. coli* vs PBS group. N = 8 in each group). (**D,E**), WISH (**D**) and calculation (**E**) of the *pu.1*^+^ myeloid progenitors in the CHT of stressed WT (214.50 ± 13.98 vs 78.10 ± 6.89 in *E. coli* vs PBS group. N = 8 in each group) or *pu.1*^*G242D/G242D*^ larvae (230.30 ± 24.54 vs 187.00 ± 17.78 in *E. coli* vs PBS group. N = 8 in each group). (**F,G**), WISH (**F**) and calculation (**G**) of *mfap4*^+^ macrophages in the CHT of challenged WT (128.80 ± 7.32 vs 75.60 ± 5.09 in *E. coli* vs the group. N = 8 in each group) or *pu.1*^*G242D/G242D*^ larvae (174.50 ± 14.03 vs 49.30 ± 3.12 in *E. coli* vs PBS group. N = 8 in each group). (**H**), The fold expansion of various cell types in both the infected WT (SB^+^: 2.24; *pu.1*^+^: 2.75; *mfap4*^+^: 1.70) or *pu.1*^*G242D/G242D*^ (SB^+^: 1.41; *pu.1*^+^: 1.22; *mfap4*^+^: 3.54) at 2 dpi. (**I**), Kaplan–Meier survival analysis of PBS- and *E. coli*-treated WT and *pu.1*^*G242D/G242D*^ embryos. The total numbers of animals used in each experiment are 100 (log-rank/Mantel–Cox statistic). Red arrows indicate WISH signals.

The function of Pu.1 in emergency myelopoiesis was explored because it presented significant increases in expression levels after infection (Fig. 5C,D). To this end, Pu.1 was functionally disrupted using either *pu.1*^*G242D/G242D*^ hypomorphic alleles or morpholinos (MOs) knockdown^41^,^42^. In agreement with previous studies^43^,^44^, the large phagocytic foci that appeared in wild type (WT) embryos (see supplementary Video S6) were detected in small numbers, and the clearance of *E. coli* was much slower in challenged Pu.1-deficient embryos (Fig. 6A; see supplementary Video S7). Furthermore, these infected embryos showed the highest mortality (Fig. 6I). Together, these results suggested the presence of defective phagocytosis and immune responses in the absence of normal Pu.1 function^43^,^44^. Next, emergency myelopoiesis in challenged *pu.1*-deficient larvae was examined. Because deficiency in Pu.1 activity resulted in obvious defects in macrophage development but an expansion of the neutrophil population in larvae during the steady state^41^, we predicted that Pu.1 was probably dispensable for emergency granulopoiesis but played critical roles in emergency monopoiesis. To test this hypothesis, emergency granulopoiesis was first examined. The results indicated that, even without the normal function of Pu.1, emergency granulopoiesis took place, as evidenced by the fact that the SB^+^ and *lyz*^+^ neutrophils and *pu.1*^+^ and *cebpα*^+^ myeloid progenitors in *pu.1*-deficient larvae expanded noticeably to the level of their counterparts in infected WT larvae at 2 dpi (Fig. 6B–E; see supplemental Figure S6A,B). The baseline of neutrophils and their progenitor population in these larvae was higher than the baseline in WT^41^ (Fig. 6C,E), indicating that the expansion potential of neutrophils after infection was smaller than that in WT (Fig. 6H). However, emergency granulopoiesis still occurred, although the intensity was not as strong as that in WT. Thus, Pu.1 was largely dispensable for emergency granulopoiesis. However, when the macrophage marker *mfap4* was checked, the *mfap4*^+^ cells that were markedly reduced in PBS-treated *pu.1*^*G242D/G242D*^ larvae presented a surprisingly drastic expansion after infection (Fig. 6F,G). Their number was even higher than that in the infected WT larvae at 2 dpi (Fig. 6G). Consistently, the expansion potential of *mfap4*^+^ cells was much higher than that in WT (Fig. 6H). Other macrophage markers, including *csf1ra* and *mpeg1*, displayed similar expansion in the *E. coli*-challenged *pu.1*^*G242D/G242D*^ larvae (see supplemental Figure S6C,D), further confirming that the macrophage lineage dramatically expanded in the emergency condition when Pu.1 was defective. Therefore, Pu.1 functioned differently during the demanding situation of monopoiesis. Although Pu.1 is essential for normal macrophage formation, this finding revealed its suppressive roles during emergency monopoiesis.

### Protective roles of Pu.1 in HSPC survival following challenge

**Figure 7:**
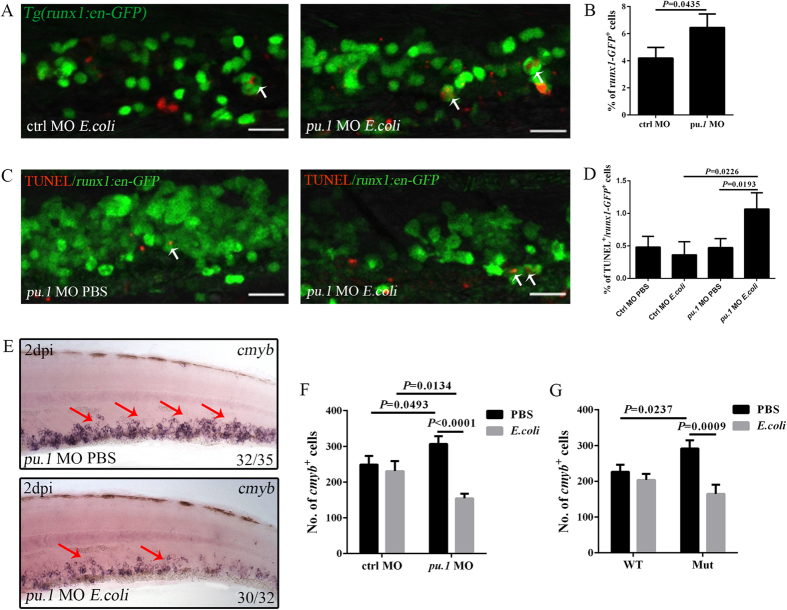
Pu.1 deficiency causes excessive HSPC apoptosis after intravenous *E. coli* (5–10 × 10^3^ cfu) infection. (**A,B**), Fluorescence images (**A**) and calculation (**B**) of runx1-GFP^+^ cells that phagocytose (white arrows) *E. coli* in the control (4.20 ± 0.79; N = 13) or *pu.1* morphant (6.46 ± 1.0; N = 11) CHT at 6 hpi. (**C**), The fluorescence images of double staining by GFP and TUNEL in the CHT of PBS or *E. coli*-treated *pu.1* morphants *Tg(runx1:en-GFP)* embryos at 2 dpi (4dpf). White arrows show the co-localization of TUNEL and GFP signals. (**D**), The percentage of TUNEL^+^/runx1-GFP^+^ in each group at 2 dpi (4 dpf) (Ctrl MO PBS: 0.48 ± 0.16; Ctrl MO *E. coli*: 0.36 ± 0.20; *pu.1* MO PBS: 0.47 ± 0.14; *pu.1*MO *E. coli:* 1.07 ± 0.25. N ≥ 8 in each group). (**E**), WISH of *cmyb* (red arrows) in the CHT of the PBS or *E. coli*-treated *pu.1* morphants at 2 dpi (4 dpf). (**F**), The numbers of *cmyb*^+^ cells in the CHT of treated control (230.80 ± 28.00 vs 248.80 ± 24.50 in *E. coli* vs PBS group. N = 10 in each group) or *pu.1* morphants (154.40 ± 13.03 vs 306.60 ± 21.68 in *E. coli* vs PBS group. N = 10 in each group). (**G**) The numbers of *cmyb*^+^ cells in the CHT of the treated WT (203.80 ± 17.06 vs 226.40 ± 20.07 in *E. coli* vs PBS group. N = 10 in each group) or *pu.1*^*G242D/G242D*^ embryos (164.80 ± 25.60 vs 291.70 ± 23.22 in *E. coli* vs PBS group. N = 10 in each group) at 2 dpi (4 dpf).

A deficiency in the efficient clearance of bacteria and the resulting greater severity of bacteraemia-like syndrome in Pu.1-deficient embryos probably increased the exposure of HSPCs to microbes in the CHT. We were interested in the reaction of the HSPCs in this scenario. To this end, *Tg(runx1:en-GFP)* larvae were treated with control and *pu.1* MOs. The response of runx1-GFP^+^ cells following bacterial challenge was investigated. Approximately 4% of the runx1-GFP^+^ cells were observed to engulf bacteria in the control group. However, this population clearly expanded in the *pu.1* morphants (Fig. 7A,B), suggesting that HSPCs could directly interact with microbes in the CHT and that during more severe infection, more HSPCs were involved. Consequently, more runx1-GFP^+^ cells underwent apoptosis, as evidenced by the increased percentage of TUNEL^+^/runx1-GFP^+^ apoptotic cells in the challenged *pu.1* morphants, and the level was higher than that detected in either the infected control embryos or the PBS-treated *pu.1* morphants (Fig. 7C,D). Therefore, Pu.1 was critical for HSPC survival following challenge. To confirm this conclusion, another HSPC marker, *cmyb,* was evaluated. In agreement with the findings in runx1-GFP^+^ HSPCs, cmyb-GFP^+^ cells underwent similar excessive apoptosis in infected *pu.1* morphants (see supplemental Figure S6E,F). However, the cmyb-GFP^+^ cells underwent similar proliferation in both the control and *pu.1* morphants treated with either PBS or *E. coli* (see supplemental Figure S6G,H) when examined using anti-pH3 antibody staining^30^, suggesting a dispensable role for Pu.1 in infection-induced HSPC proliferation. To accurately present the data, the *cmyb*^+^ cells were quantified. The *cmyb*^+^ cell number was markedly lower in the treated *pu.1* morphants than in the control group at 2 dpi (Fig. 7E,F), which was consistent with their increased apoptosis (see supplemental Figure S6E,F). A similar phenomenon was recapitulated in *pu.1*^*G242D/G242D*^ embryos, although the reduction of *cmyb*^+^ cells was less drastic in *pu.1*^*G242D/G242D*^than in *pu.1* morphants(Fig. 7F,G). This result is likely a consequence of the partial disruption of Pu.1 activity in *pu.1*^*G242D/G242D*^compared with the more severe disruption resulting from a high dose of *pu.1* MOs^41^. Thus, Pu.1 was essential for the efficient clearance of microbes, which in turn prevented over-exposure of HSPCs to microbes. This procedure is quite important for HSPC homeostasis after *E. coli* challenge.

The reduction of HSPCs but expansion of myeloid progenitors in the challenged Pu.1-deficient larvae appeared to be contradictory, which led us to suspect that *E. coli* affects HSPCs and myeloid progenitors in distinct manners. To verify our hypothesis, co-staining of cmyb-GFP and *pu.1* was performed in *Tg(cmyb:eGFP).* In the control group treated with PBS, the majority of the cmyb-GFP^+^ cells expressed *pu.1* signals, resulting in a small ratio (approximately 23%) of *pu.1*^+^-only myeloid progenitors (see supplemental Figure S7). However, upon challenge, the *pu.1*^+^-only cell population increased dramatically to approximately 41% (see supplemental Figure S7), suggesting that the *pu.1*^+^-only myeloid progenitors themselves underwent notable expansion upon challenge. Concordantly, in *E. coli*-challenged *pu.1* morphants, *pu.1*^+^-only myeloid progenitors showed drastic expansion, but cmyb-GFP^+^ HSPCs showed a clear reduction when compared with their control counterparts (see supplemental Figure S7). Consequently, the percentage of *pu.1*^+^-only myeloid progenitors increased to approximately 52%, which was much higher than that of the other groups (see supplemental Figure S7). Therefore, the HSPCs and myeloid progenitors separately responded to the microbes, and Pu.1 deficiency led to a reduction in HSPCs but showed a limited influence on the expansion of the myeloid progenitors.

## Discussion

Taking advantage of the optical transparency of zebrafish larvae, an emergency myelopoiesis model was established through direct injection of Dsred^+^ *E. coli*^13^,^23^ into the circulatory system. Although intravenous injection of microbes has been employed by several groups^10^,^11^,^12^,^13^, infection-induced myelopoiesis has rarely been the experimental focus. Recently, Kathryn E. Crosier’s group dissected the role of the Cebpβ-Nos2a pathway in demand-adapted emergency granulopoiesis by injecting GFP^+^ *Salmonella* into the brains of larvae. In that study, emergency granulopoiesis was achieved by sacrificing lymphopoiesis, and HSPCs clearly increased under this condition; by contrast, macrophages showed no notable increase in the trunk region^10^. This work facilitated the initiation of research investigating infection-induced hematopoiesis using larval zebrafish^45^. However, the reaction of hematopoietic cells to systemic infection, particularly when HSPCs directly encountered microbes in CHT, has not been addressed. In our study, direct inoculation of microbes into the zebrafish bloodstream led to the expansion of both macrophages and neutrophils. Thus, emergency monopoiesis, in addition to emergency granulopoiesis, occurred with the use of this method, which could serve as a good supplementary assay to study emergency myelopoiesis^10^.

In contrast to locally infected larvae^10^, direct injection of bacteria into the circulation led to the development of a bacteraemia-like syndrome, which caused immediate and significant participation of macrophages and neutrophils in phagocytosis and digestion of microbes^13^,^23^,^27^. The intensive involvement of myeloid phagocytes led to their increased apoptosis and quick exhaustion, which was probably the cue for their subsequent expansion^46^. The expanded macrophages and neutrophils in the challenged larvae were probably of different origins, as suggested by the data obtained for *runx1*^*w84x *24^. Almost no neutrophils were found in *runx1-*deficient larvae, regardless of whether they were challenged by microbes, thus supporting a *runx1*-dependent definitive hematopoietic origin of granulopoiesis under both physiological and stressed conditions. The macrophages slightly expanded in the steady state^41^, and emergency monopoiesis occurred normally in the *runx1* mutant. This result suggested that the macrophages at this stage were largely generated from primitive hematopoiesis, which occurred independently of *runx1*. A recent study has demonstrated that the microglia, a subtype of macrophages in the brain, mainly originates from primitive myelopoiesis throughout the larval period^47^. In another study, a mutant fish line with compromised definitive hematopoiesis showed a limited influence on macrophages at later larval stages^48^. Thus, it is possible that larval macrophages have a largely primitive origin.

The HSPCs directly interacted with the microbes in the CHT in our assay. And their response was dependent on the bacterial burden. A relatively lower volume of *E. coli* led to a moderate expansion of HSPCs, which is consistent with previous reports^10^,^33^,^34^. However, excessive stress caused by a large microbial burden resulted in a drastic exhaustion of HSPCs. The distinct reactions of the HSPCs to different bacterial burdens were probably related to the severity of the direct exposure of HSPCs to the microbes and the overproduction of pro-inflammatory cytokines. Because a higher dose of *E. coli* would overcome the clearance by phagocytes and lead to the production of excessive levels of pro-inflammatory cytokines, it can be inferred that the longer and stronger influence of microbes on HSPCs in the CHT and the overproduction of pro-inflammatory cytokines probably facilitated their apoptosis. This hypothesis was further supported by the reduction of HSPCs in the infected Pu.1-deficient embryos. The functional defects of the macrophages in Pu.1-deficient embryos resulted in the slower clearance of *E. coli*^43^,^44^. Consequently, the interaction between HSPCs and pathogens was prolonged. Concordantly, increased apoptosis of HSPCs occurred, overcoming the cell proliferation and leading to a reduction of cell numbers. The increased apoptosis was correlated with excessive production of inflammatory factors, including IFNγ and TNFα (see supplemental Figure S6E). Appropriate levels of IFNγ and TNFα are essential for the activation of HSPC proliferation^5^,^35^,^36^,^37^. However, their overproduction causes rapid HSPC apoptosis^5^,^6^,^38^,^39^. Thus, the drastically altered levels of IFNγ and TNFα were probably responsible for the increased apoptosis of HSPCs.

Pu.1 is indispensable in the commitment of myeloid cells^41^,^49^,^50^ and in leukaemogenesis^51^,^52^. However, its function in infection-induced emergency myelopoiesis had not been addressed. Taking advantage of *pu.1*^*G242D/G242D*^ and morpholino-mediated functional disruption, the roles of Pu.1 in infection-induced emergency myelopoiesis were carefully dissected. Surprisingly, compared with the insensitive expansion of neutrophils, macrophages with significant physiological shortcomings in the presence of defective Pu.1 presented drastic expansion after infection, and their numbers quickly exceeded the values determined in infected WT embryos. Pu.1 seemed to be an inhibitory regulator for the infection-induced expansion of macrophages. Thus, it played distinct roles in physiological and emergency monopoiesis. A previous study has demonstrated that Cebpβ plays different roles in physiological and emergency granulopoiesis because its deficiency leads to ineffective emergency granulopoiesis, although it is dispensable for the normal development of neutrophils^2^,^10^,^53^. The data obtained for *cebpβ* and *pu.1* suggested that the regulatory networks underlying emergency myeloid cell development differed from that utilised in the steady state. Therefore, elucidation of the mechanisms responsible for emergency myelopoiesis is an interesting topic for further investigation.

## Methods

### Ethics statement

All experimental protocols were approved by the School of Life Sciences, Southwest University (Chongqing, China), and the methods were carried out in accordance with the approved guidelines. The zebrafish facility and study were approved by the Institutional Review Board of Southwest University (Chongqing, China). Zebrafish were maintained in accordance with the Guidelines of Experimental Animal Welfare from Ministry of Science and Technology of People’s Republic of China (2006) and the Institutional Animal Care and Use Committee protocols from Southwest University (2007).

### Fish lines

AB, *pu.1*^*G242D*41^, *runx1*^*w84x *24^, *Tg(runx1:en-GFP)*^31^, *Tg(cmyb:eGFP)*^32^*, Tg(coro1a:eGFP)*^29^, *Tg(mpeg1:eGFP)* and *Tg(lyz:eGFP)*^*nz11726*^ strains were used and maintained under standard conditions.

### Generation of *Tg(mpeg1:eGFP)* lines

4.1-kb DNA sequence upstream of the *mpeg1* translation start site amplified with the primers 5′- ACATGCATATCTTGCAGTATA-3′/5′- GATCGCCAGATGGGTGTTTT-3′ was used as a promoter to drive eGFP expression in the pTol2 vector. The pTol2-mpeg1-eGFP construct was injected into the wild-type fish embryos at one-cell stage. The embryos with an appropriate GFP expression were selected and raised to adults. The founder lines were identified based on their eGFP expression pattern.

### Phagocytosis assays and time-lapse live imaging

The Dsred-labeled *E. coli*^23^ were cultured as previously described^43^. The cultured *E. coli* were collected in filter-sterilized PBS prior to the injection. To quantify the burdens, the volume of *E. coli* for injection was added to 1 ml LB and then plated at 1:10 and 1:100 dilutions on LB agar supplemented with 50 mg/ml kanamycin. Colonies were counted in plates incubated at 37 °C overnight to quantify the actual infection doses. The *E. coli* volume of each concentration was then microinjected into the circulation of each anesthetized embryo. The injected embryos were anesthetized, mounted in 1% agarose, and subsequently imaged under an LSM700 confocal microscope (Carl Zeiss) (X20 objective). Images were captured every 5 min, extracted, and converted into a movie using ZEN2012 software. Movie Maker was used to create the movies.

### WISH and Sudan Black (SB) staining

Antisense RNA probes were prepared according to the standard protocol. The following digoxigenin-labeled antisense probes were used: *cmyb, pu.1, cebpα, csf1ra, mpx, cebp1, lyz, mpeg1, mfap4, rag1, ccr9a, αe1-globin* and *gata1*. Single-colour whole-mount *in situ* hybridization (WISH) was performed^24^. Sudan black (Sigma, 380B) solution was used to treat the fixed embryos^28^. The signals were observed under SteREO Discovery.V20 microscope (Carl Zeiss).

### Double staining for RNA (*pu.1*) and protein (GFP)

The double staining process was mainly performed according to a previous study^43^. Briefly, WISH staining of *pu.1* was first developed with Cy3 tyramide (PerkinElmer Life and Analytical Sciences). Afterward, the embryos were washed and incubated with goat anti-GFP antibody (1:400, 4 °C, overnight) (Abcam, ab6658) and visualized with Alexa Fluor 488 donkey anti–goat secondary antibodies (1:400, 4 °C, overnight) (Invitrogen).

### Double fluorescence immunohistochemistry staining and terminal deoxynucleotidyl transferase dUTP nick-end labeling (TUNEL)

Double fluorescence immunohistochemistry staining of larvae was performed^24^. For eGFP and pH3 double staining, *Tg(cmyb:eGFP)* embryos treated with control or *pu.1* morpholinos^41^ were fixed in 4% paraformaldehyde at the desired stages. The fixed embryos were incubated with primary rabbit anti–phospho-histone H3 (1:250, 4 °C, overnight) (pH3; Santa Cruz Biotechnology, sc-8656-R) and goat anti-GFP (1:400, 4 °C, overnight) (Abcam, ab6658) antibodies according to the manufacturer’s protocol and subsequently stained with Alexa Fluor 647 anti–rabbit and Alexa Fluor 488 anti–goat secondary antibodies (Invitrogen). For the TUNEL assays, the *in situ* cell death detection kit, TMR Red (Roche 12156792910), was applied. The staining process was performed as indicated in the protocol. All fluorescence images were obtained using an LSM700 confocal microscope (Carl Zeiss).

### Differentially expressed gene (DEG) analysis

To explore the molecules involved after infection, *E. coli*-treated embryos were selected at 6 hpi, 24 hpi and 4 dpi. Their total RNA was extracted for deep sequencing by the Biomarker Company, Beijing. The differentially expressed genes (DEGs) between any 2 samples were identified based on the following two criteria. 1) The expression value (FPKM) of the DEG must be larger than 1 in both samples, which indicates that the gene is active in these samples and that the detected expression values are not caused by background noise (for example, read mismatching or multi-hit alignment). 2) The variation in gene expression between the two conditions should be larger than 2-fold. Based on these two criteria, we identified 1441 DEGs in at least one comparative case (see Supplementary Table S1 and S2). Gene set enrichment analyses were performed for the functional annotation of the DEGs. Functional annotation tools in DAVID Bioinformatics Resources^54^ were used to conducted these analyses.

### Real-time quantitative polymerase chain reaction (qPCR)

The total RNA of the infected embryos at different time points was isolated for the qPCR^55^. Each sample was tested in triplicate. Elongation factor 1α (ef1α) expression was measured and used to normalize signals for each queried transcript using the ΔΔCt method. The primers used (5′-3′): TLR5a-GAGGTGCCAAAGATTTCCACTTAC/TGGTGCATCAGGATGAGGACT;mpx-CCTCAACGACAGCACTCTGA/TACTCCAGGTAGGGTTGAGCA; il1b-CCCCAATCCACAGAGTTT/TTCACTTCACGCTCTTGG; mmp9-CATTAAAGATGCCCTGATGTATCCC/AGTGGTGGTCCGTGGTTGAG; cmyb-TTTCTACCGAATCGAACAGATG/CAATCACCCGTTGGTCTTCT; irf8-CCATTTTCAAAGCGTGGGCA/CTGGCACAATCCGGTACACT; csf3r-TGAAGGATCTTCAACCACAC/GGGAATTATAGGCCACAAAC;pu.1-AGAGAGGGTAACCTGGACTG/AAGTCCACTGGATGAATGTG.;ifng1-2-CTATGGGCGATCAAGGAAAA/CTTTAGCCTGCCGTCTCTTG. Other inflammatory cytokines were designed as according to a previous study^37^.

### Quantification, calculation and statistical methods

To quantify the WISH, SB, fluoresce and immunohistochemistry signals, the positive signals on the images of the larval CHT regions (WISH and SB: 10 somites; fluoresce and immunohistochemistry: 6 somites) were manually counted. All the quantified data were double confirmed and analysed by GraphPad Prism 6. Student’s *t* test (one tailed) was mainly used (Mean ± SEM). Survival was calculated with Kaplan-Meier calculations.

## Additional Information

**How to cite this article**: Hou, Y. *et al*. Systemic inoculation of *Escherichia coli* causes emergency myelopoiesis in zebrafish larval caudal hematopoietic tissue. *Sci. Rep.* **6**, 36853; doi: 10.1038/srep36853 (2016).

**Publisher’s note**: Springer Nature remains neutral with regard to jurisdictional claims in published maps and institutional affiliations.

## Supplementary Material

Supplementary Video S1

Supplementary Video S2

Supplementary Video S3

Supplementary Video S4

Supplementary Video S5

Supplementary Video S6

Supplementary Video S7

Supplementary Information

Supplementary Table S2
