# Supplementary material for: Systemic inoculation of Escherichia coli causes emergency myelopoiesis in zebrafish larval caudal hematopoietic tissue
Source: Sci Rep. 2016 Nov 11;6:36853. doi: 10.1038/srep36853 (PMC5105072; doi:10.1038/srep36853)
Supplement: Supplementary Information [file srep36853-s8.doc]

**Supplementary Information**

**Systemic inoculation of *Escherichia coli* causes emergency myelopoiesis in zebrafish larval caudal hematopoietic tissue**

Yuelan Hou^1,*^, Zhen Sheng^2,*^, Xiaobing Mao^1^, Chenzheng Li^1^, Jingying Chen^1^, Jingjing Zhang^3^, Honghui Huang^1^, Hua Ruan^1^, Lingfei Luo^1^ and Li Li^1,#^

^1^Key Laboratory of Freshwater Fish Reproduction and Development, Ministry of Education, Key Laboratory of Aquatic Science of Chongqing, Laboratory of Molecular Developmental Biology, School of Life Sciences, Southwest University, Chongqing, 400715, China

^2^Bioinformatics Department College of Life Science and Biotechnology, Tongji University, 200092, China

^3^Affiliated Hospital of Guangdong Medical College, Zhanjiang, Guangdong, 524001, China

^*^These authors contributed equally to this work.

^#^Correspondence: [alisir@swu.edu.cn](mailto:alisir@swu.edu.cn);.

Phone: +86-23-68254946

**
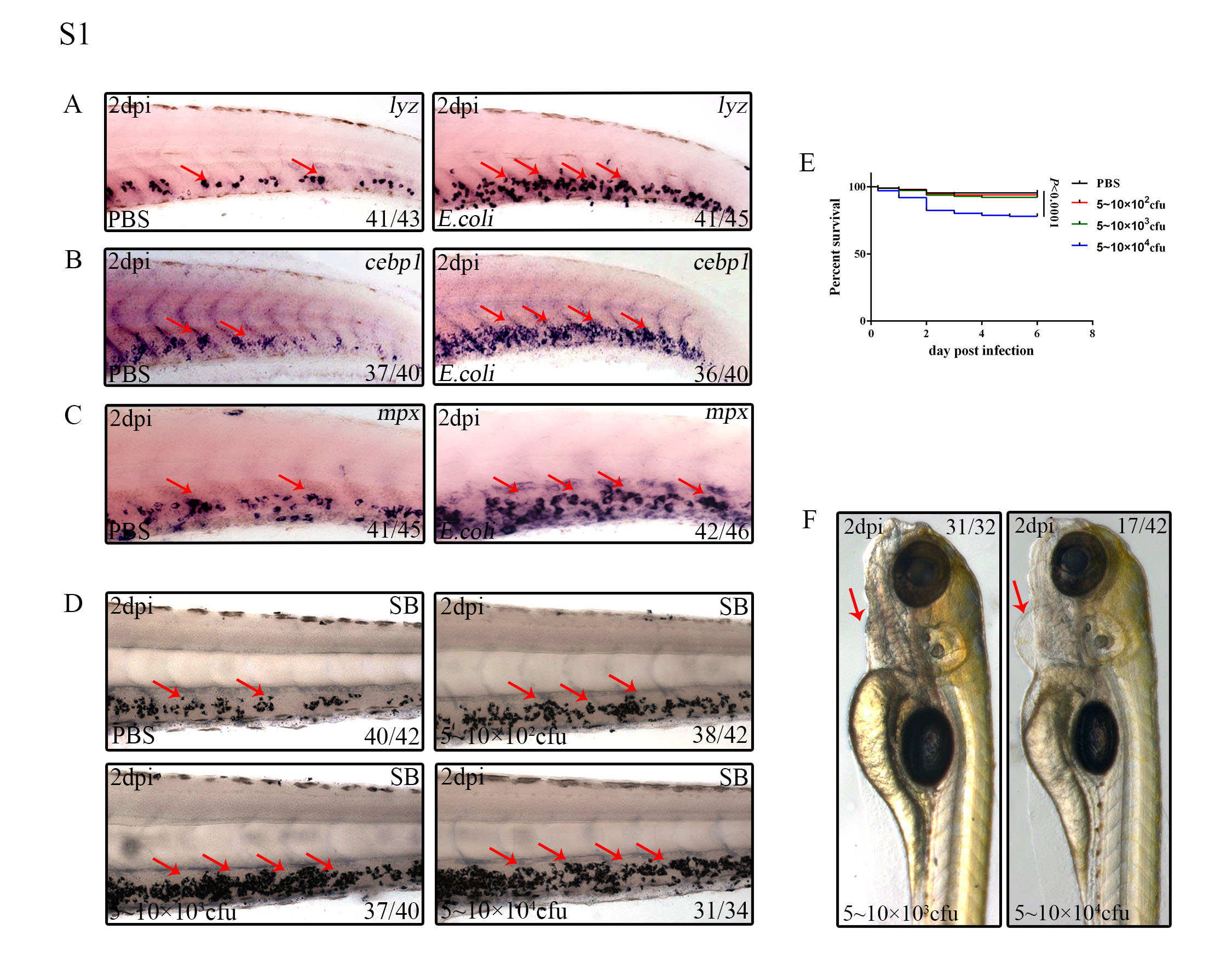
Figure S1. Reaction of neutrophils to different volumes of intravenously injected *E. coli*.**

(A-C), WISH of *lyz* (A), *cebp1* (B) and *mpx* (C) in *E. coli* (5-10x10^3^ cfu) or PBS-treated embryos at 2 dpi (4 dpf). Red arrows in each panel denote WISH signals. (D), SB staining of larval CHT treated with various volumes of *E. coli*. Red arrows indicate SB signals. (E), Kaplan–Meier survival analysis of embryos treated with PBS or various volumes of *E. coli*. One hundred embryos are assessed in each group. (F), The morphological appearance of larvae challenged with 5-10x10^3^ cfu and 5-10x10^4^ CFU *E. coli* at 2 dpi (4 dpf). The red arrows indicate pericardial edema.


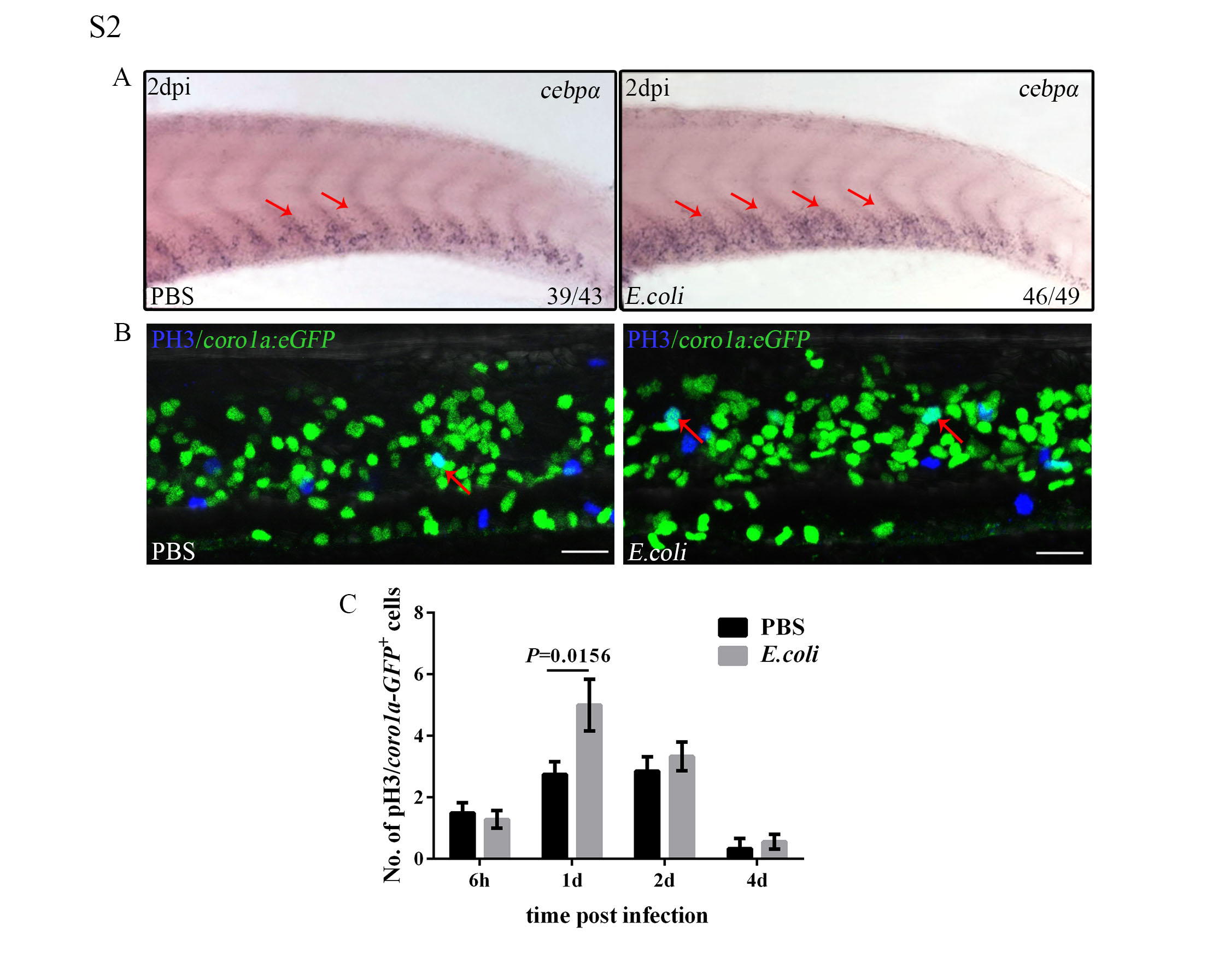


**Figure S2. Expansion and proliferation of myeloid cells following intravenous *E. coli* (5-10x10^3^ cfu) challenge.**

(A), WISH of *cebpα* in PBS or *E. coli*-treated embryos at 2 dpi (4 dpf). Red arrows indicate WISH signals. (B), The fluorescence images indicate double staining of coro1a-GFP and pH3 in the CHT of PBS or *E. coli*-treated *Tg(coro1a:eGFP)* embryos at 2 dpi (4 dpf). Red arrows indicate the co-staining of pH3 and coro1a-GFP. Scale bars, 20 µm. (C), The number of pH3^+^/coro1a-GFP^+^ cells in both PBS and *E. coli*-treated *Tg(coro1a:GFP)* CHT at different time points (1.30±0.29 vs 1.50±0.33; 5.00±0.85 vs 2.80±0.41; 3.30±0.47 vs 2.90±0.46; 0.60±0.24 vs 0.30±0.33; in *E. coli* vs PBS group at each time point. N ≥ 8 in each group).

**
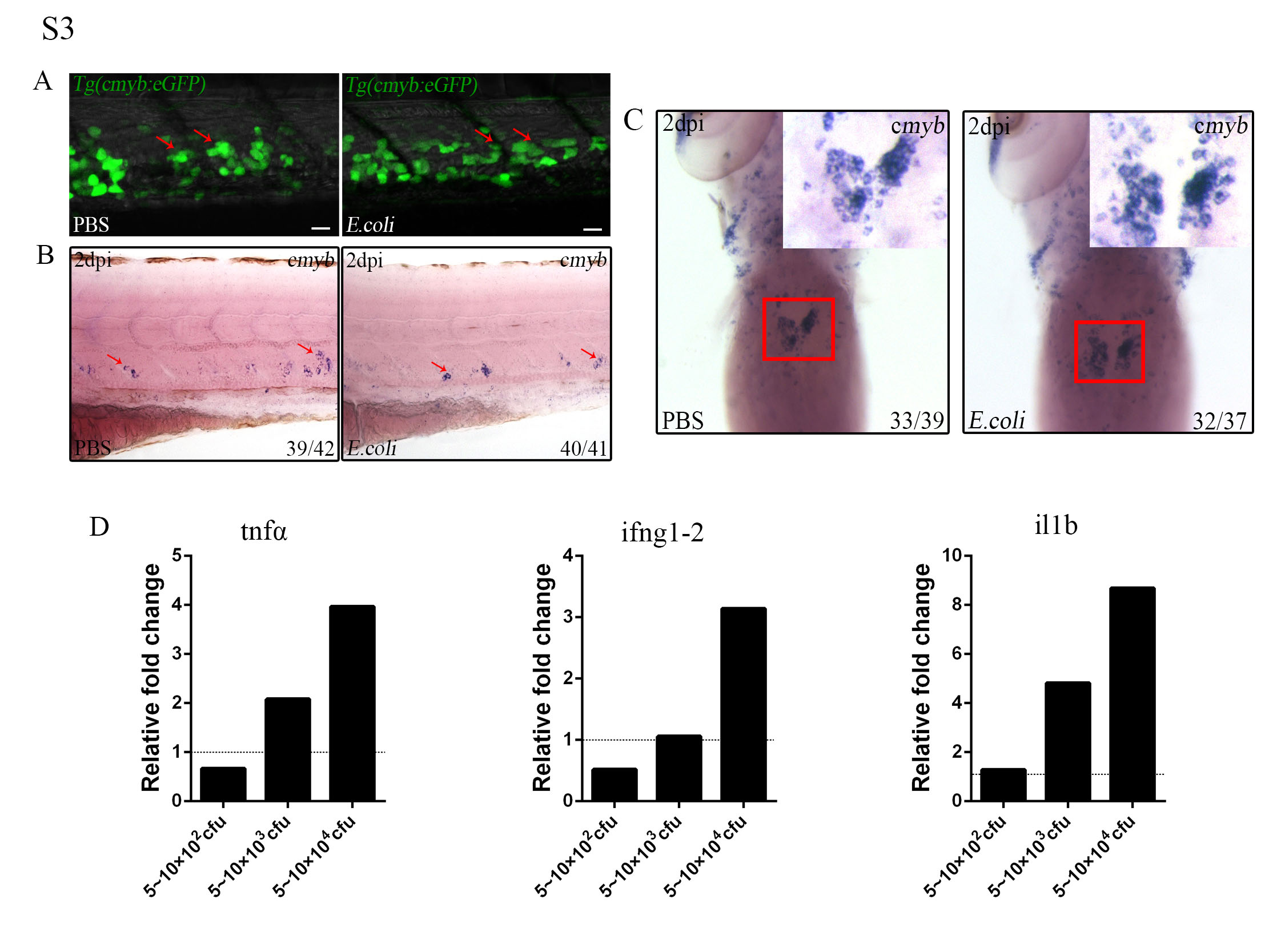
Figure S3. Homeostasis of HSPCs upon intravenous treatment of 5-10x10^3^ cfu *E. coli*.**

(A), Fluorescence images showing that cmyb-GFP^+^ cells do not present obvious alterations in *E. coli*-infected compared with PBS-treated *Tg(cmyb:eGFP)*. Red arrows denote GFP^+^ signals. Scale bars, 20 µm. (B-C), WISH of *cmyb* in the trunk (B) and kidney (C) of *E. coli* or PBS-treated embryos at 2 dpi (4 dpf). Red arrows in (B) indicate WISH signals. The small panel inserts in (C) are magnified images of the boxed region in the kidney. (D) qPCR showing the expression level of *tnfα, ifng1-2,* and *il1b* in larvae treated with different volumes of *E. coli* at 2 dpi (4 dpf).

**
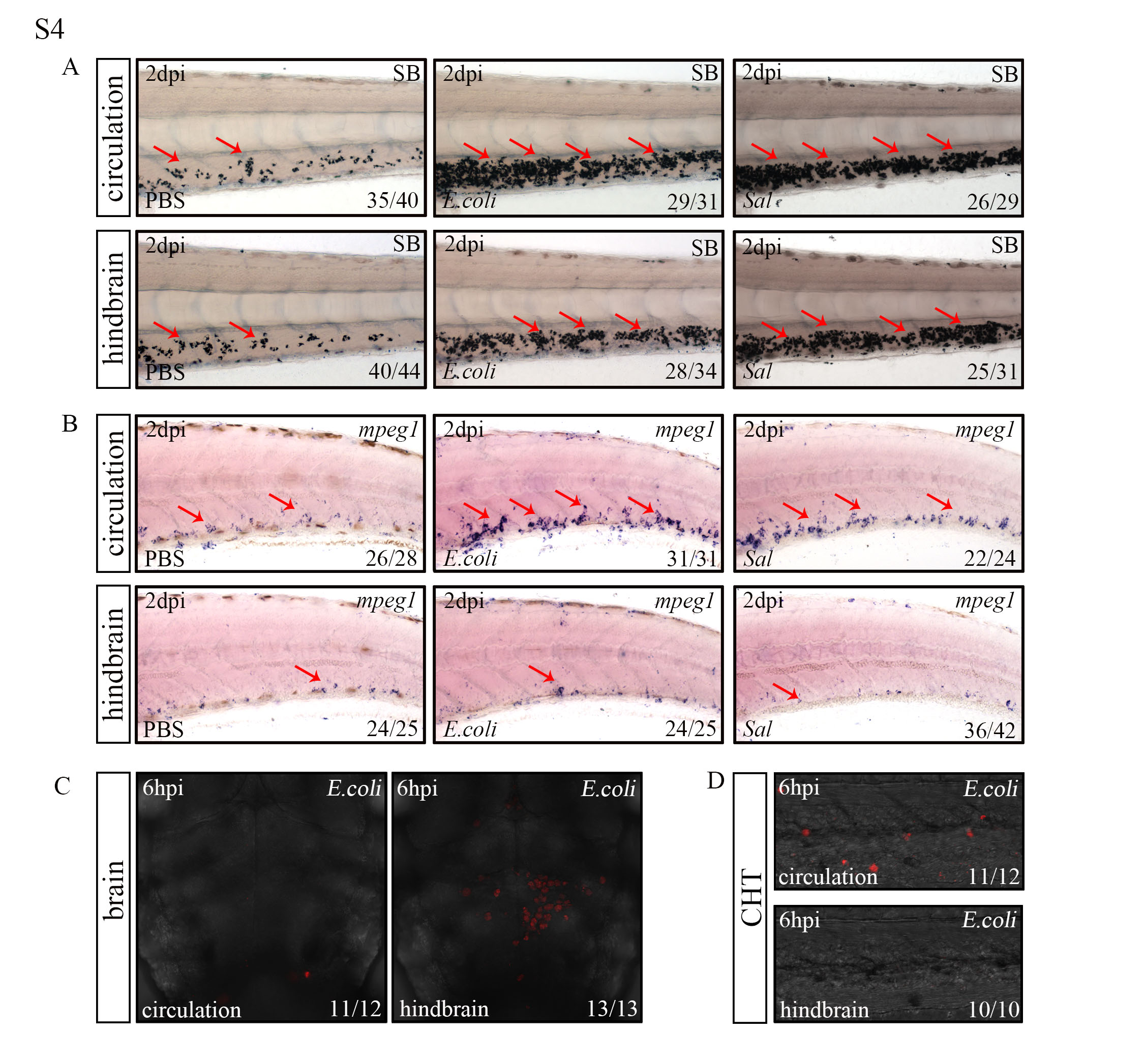
Figure S4. Expansion of macrophages following direct injection of microbes into the circulation.**

(A), Injection of *E. coli* or *salmonella typhimurium* via either the circulation or hindbrain induces similar expansion of SB*^+^* neutrophils in a larval CHT compared with PBS treatment at 2 dpi (4 dpf). (B), WISH present a drastic expansion of *mpeg1^+^* macrophages in the larval CHT infected via the circulation but not the hindbrain. Red arrows indicate SB*^+^* (A) and *mpeg1 ^+^* (B) signals. (C-D), Fluorescence images show the distribution of Dsred^+^ *E. coli* in the brain (C) and CHT (D) of larvae infected through either the circulation or hindbrain.

**
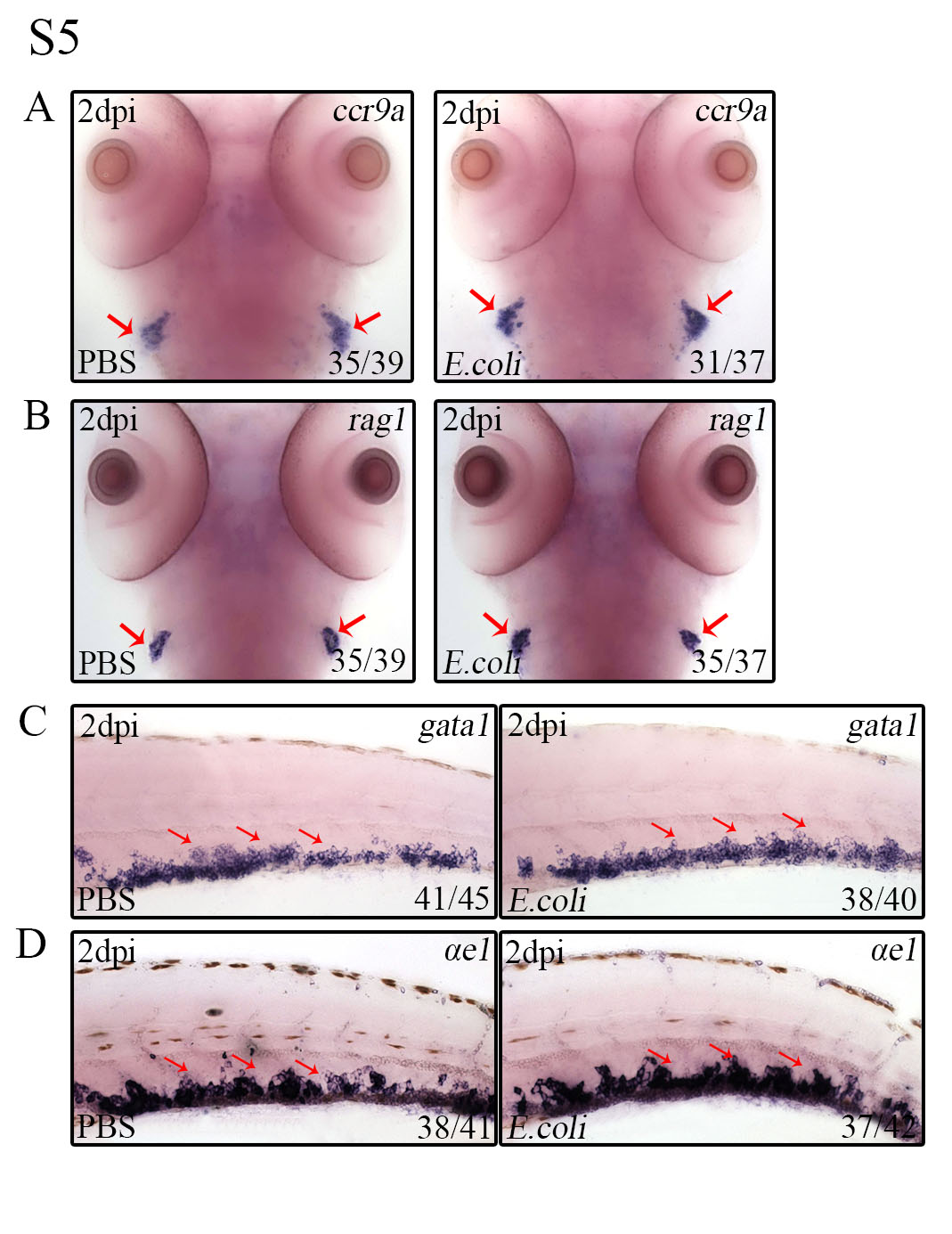
**

**Figure S5. No obvious alterations in other lineages in the embryos after intravenous injection of *E. coli* (5-10x10^3^ cfu).**

(A-D), WISH indicate similar levels of *ccr9a^+^* (A) and *rag1^+^*(B) T lymphoid cells in the thymus as well as the *gata1^+^* (C) and *αe1^+^* (D) erythrocytes in the CHT of PBS and *E. coli*-treated embryos at 2 dpi (4 dpf). The red arrows in each panel indicate the WISH signals.

**
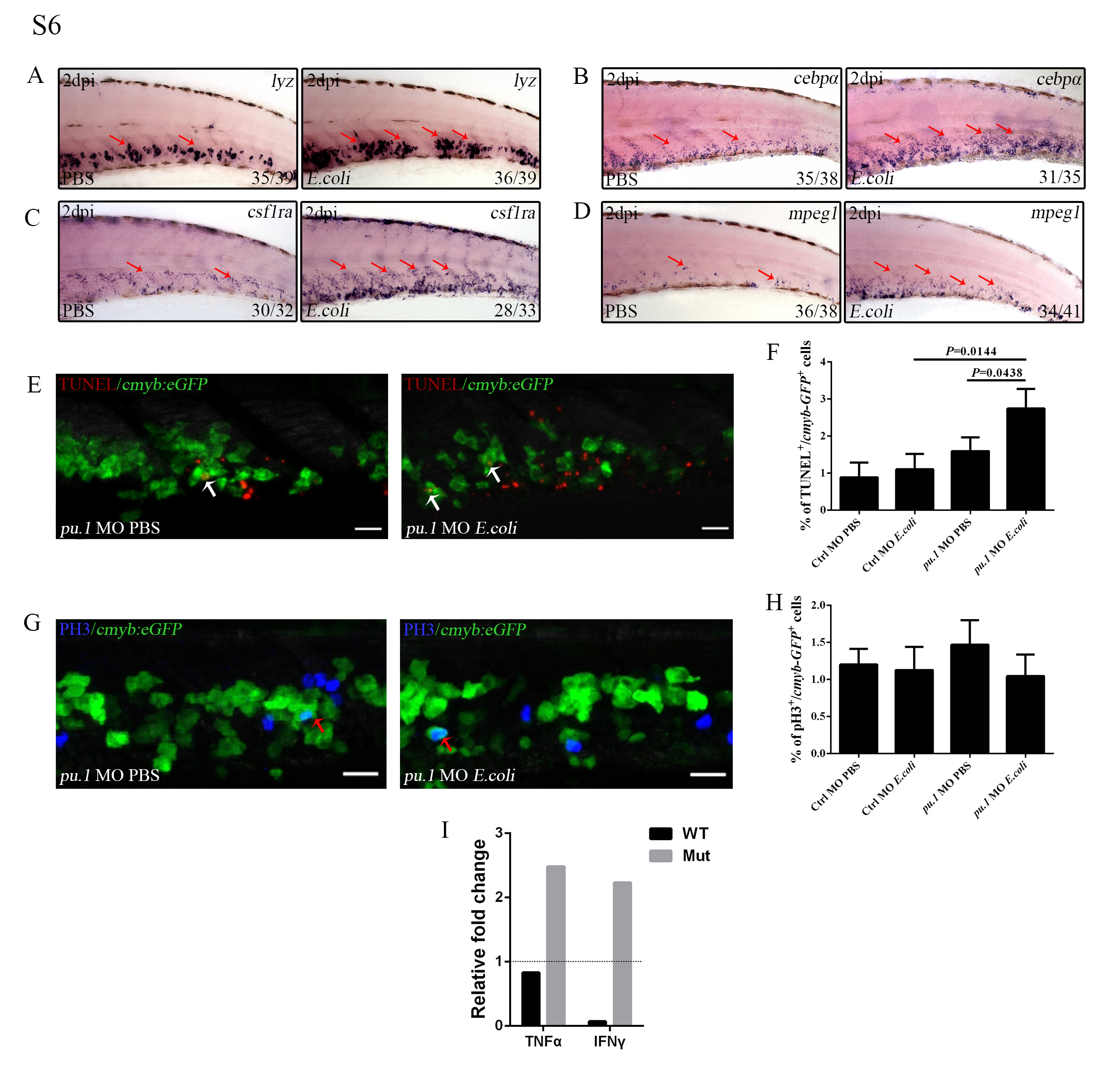
Figure S6. The hematopoietic reaction in Pu.1-deficient embryos after intravenous injection of *E. coli* (5-10x10^3^ cfu).**

(A-D), WISH of *lyz* (A), *cebpα* (B) *csf1ra* (C) and *mpeg1* (D) in the CHT of PBS or *E. coli*-treated *pu.1^G242D/ G242D^* embryos at 2 dpi (4 dpf). Red arrows show WISH signals. (E), Fluorescence images show double staining of GFP and TUNEL in the CHT of the PBS or *E. coli*-treated *pu.1* morphant *Tg(cmyb:eGFP)* embryos at 2 dpi (4 dpf). White arrows show the co-localization of TUNEL and GFP signals. Scale bars, 20 µm. (F), The percentage of TUNEL^+^/cmyb-GFP^+^ in *E. coli*-challenged *pu.1* morphants is much higher than that in either the PBS-treated *pu.1* morphants or *E. coli*-challenged control embryos at 2 dpi (4 dpf) (Ctrl MO PBS: 0.90±0.40; Ctrl MO *E. coli*: 1.10±0.42; *pu.1* MO PBS: 1.60±0.37; *pu.1*MO *E.coli:* 2.70±0.53. N≥9 in each group). (G), Fluorescence images showing double staining of cmyb-GFP and pH3 in the CHT of PBS and *E. coli*-treated *pu.1* morphant *Tg(cmyb:eGFP)* embryos at 2 dpi (4 dpf). Red arrows indicate co-staining of pH3 and cmyb-GFP. Scale bars, 20 µm. (H), The data showing the percentage of pH3^+^/cmyb-GFP^+^ cells in the cmyb-GFP^+^ population in various groups (Ctrl MO PBS: 1.20±0.21; Ctrl MO *E. coli*: 1.10±0.31; *pu.1* MO PBS: 1.50±0.33; *pu.1*MO *E. coli:* 1.00±0.29. N≥9 in each group). (I), qPCR showing the drastic elevation of TNF*α* (2.48 vs 0.83) and IFNγ (2.23 vs 0.07) in the infected *pu.1^G242D/ G242D^* compared with WT counterpart at 2 dpi (4 dpf).

**
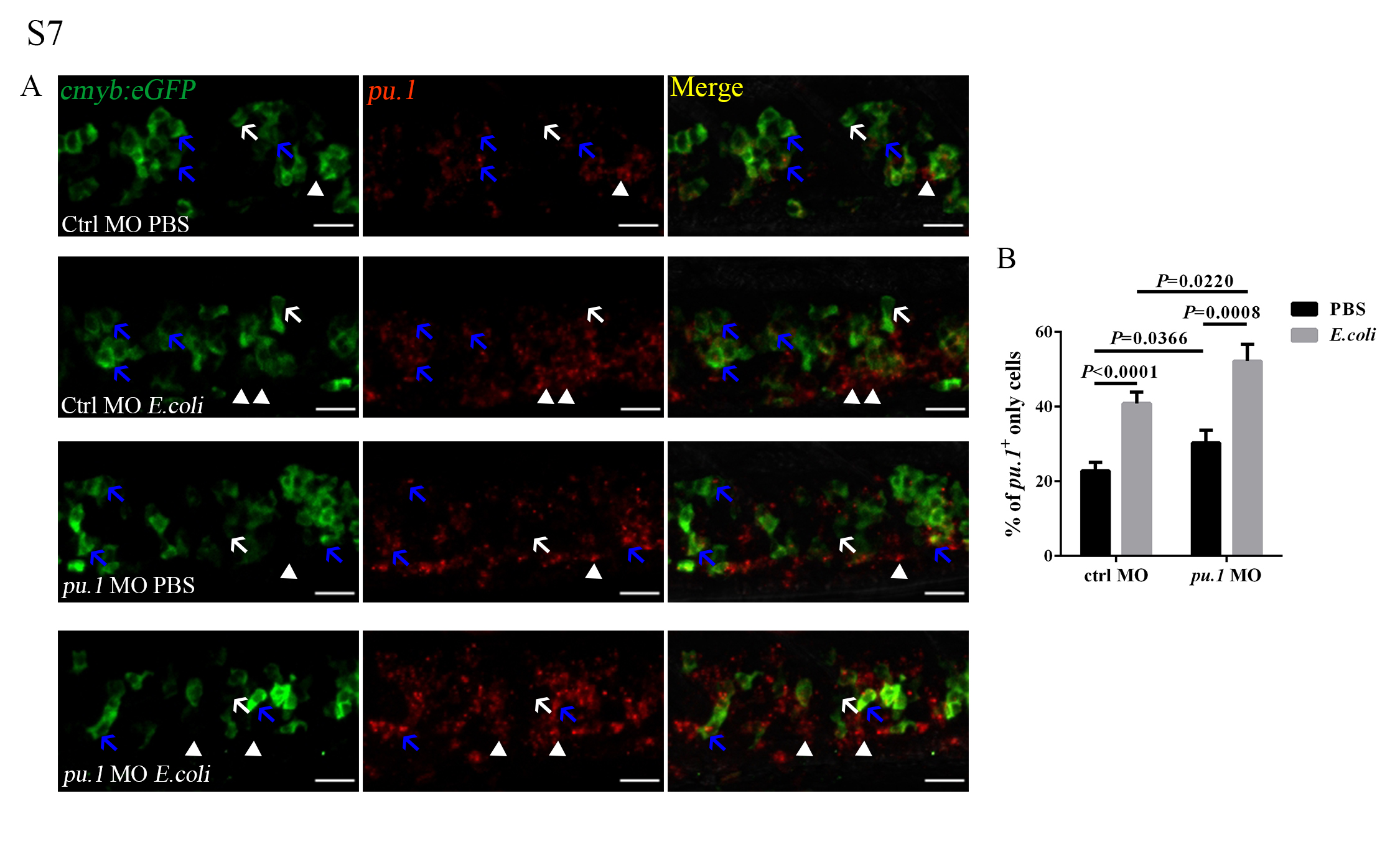
Figure S7. Myeloid progenitor expansion but HSPC reduction in *pu.1* morphants after intravenous *E. coli* (5-10x10^3^ cfu) challenge.**

(A), Fluorescence images showing double staining of the cmyb-GFP and *pu.1* in the PBS or *E. coli*-treated control and *pu.1* morphant *Tg(cmyb:eGFP)* larvae at 2 dpi (4 dpf). The white arrows indicate cmyb-GFP^+^ only HSPCs. The blue arrows denote cmyb-GFP^+^ cells that began to express low levels of *pu.1*. The white arrowheads indicate *pu.1^+^* only myeloid progenitors. Scale bars, 20 µm. (B), The percentage of *pu.1^+^* only myeloid progenitors in (A) (ctrl MO PBS: 22.79 ± 2.32, ctrl MO *E. coli*: 40.90 ± 3.02, *pu.1* MO PBS: 30.30 ± 3.37, *pu.1*MO *E. coli*: 52.22 ± 4.53; N ≥ 9 in each group).

**Table S1. General statistical information of RNAseq data**

|  | 6hpi PBS | 6hpi *E.coli* | 24hpi PBS | 24hpi *E.coli* | 4dpi PBS | 4dpi *E.coli* |
| --- | --- | --- | --- | --- | --- | --- |
| Total Reads | 48700618 (100%)^a^ | 45507828 (100%) | 50517922 (100%) | 53703324 (100%) | 53808358 (100%) | 48351206 (100%) |
| mapped Reads | 32087661 (66%) | 30567204 (67%) | 32519462 (64%) | 36048988 (67%) | 37812614 (70%) | 33532701 (69%) |
| Uniquely mapped reads | 30918488 (64%) | 29467009 (65%) | 31341636 (62%) | 34812006 (65%) | 36477513 (68%) | 32383505 (67%) |
| Multiple mapped reads | 1169173 (2%) | 110019  (2%) | 1177826 (2%) | 1236982 (2%) | 1335101 (2%) | 1149196 (2%) |
| Paired mapped reads | 25376375 (52%) | 24996024 (55%) | 25886539 (51%) | 29214679 (54%) | 31638827 (59%) | 27994555 (58%) |
| Single mapped reads | 6031542 (12%) | 5118429 (11%) | 6039427 (12%) | 6234359 (12%) | 5710744 (11%) | 5138169 (11%) |

**Table S2. The detailed information of differentially expressed genes (DEGs) induced by bacterial infection at different time points (6hpi, 1dpi, 4dpi).**

A. downregulated DEGs after infection. B. upregulated DEGs after infection. In each sheet of table, each row is colored according to the subgroup of the gene in Figure 5A or Figure 5B. Khaki: the gene upregulated or downregulated at all the three time points. Violet: the gene upregulated or downregulated only at time points of 6hpi and 1dpi. Green: the gene upregulated or downregulated only at time points of 6hpi and 4dpi. Pale red: the gene upregulated or downregulated only at time points of 1dpi and 4dpi. Iceblue: the gene only upregulated or downregulated at 6hpi. Red: the gene only upregulated or downregulated at 1dpi. Yellow: the gene only upregulated or downregulated at 4dpi.

**Video S1. The reaction of macrophages in a larval CHT after intravenous injection of *E. coli* (5-10x10^3^ cfu).**

This video shows time-lapse confocal fluorescence imaging of the CHT region of an *E. coli-*infected *Tg(mpeg1:eGFP)* zebrafish larva from 30 minutes to 6 hours post-intravenous infection (1 frame = 5 min). The *E. coli* circulate in the vessels after injection. The mpeg1-GFP^+^ macrophages quickly interact and engulf large amounts of bacteria (white stars). As the result, several large foci appear. Gradually, the mpeg1-GFP^+^ macrophages with huge bacterial burdens undergo cell death, resulting in weak and even the loss of fluorescence (white arrowheads). The dying macrophages are quickly engulfed by the adjacent macrophages (white arrowheads). The video shows only one confocal plane; selected frames are shown in Figure 1D, which also shows images from other z-planes of the same time-lapse confocal series. Time is indicated in hours and minutes. Scale bars, 20 µm.

**Video S2. The reaction of neutrophils in a larval CHT after intravenous injection of *E. coli* (5-10x10^3^ cfu).**

This video shows time-lapse confocal fluorescence imaging of the CHT region of an *E. coli-*infected *Tg(lyz:eGFP)* zebrafish larva from 30 minutes to 6 hours post-intravenous infection (1 frame = 5 min). The *E. coli* circulat in the vessels after injection. Initially, lyz-GFP^+^ neutrophils do not phagocytose the bacteria but aggregate large amounts of bacteria on their surface (white arrowheads). Subsequently, the adhesive bacteria are phagocytosed by neutrophils (white arrowhead). However, the volume of *E. coli* engulfed by the lyz-GFP^+^ neutrophils is less than that of macrophages, and the foci were smaller (white arrowhead). The video shows only one confocal plane; selected frames are shown in Fig 1E, which also shows images from other z-planes of the same time-lapse confocal series. Time is indicated in hours and minutes. Scale bars, 20 µm.

**Video S3. The behavior of lyz-GFP^+^ neutrophils in the CHT after PBS treatment.**

This video shows time-lapse confocal fluorescence imaging of the CHT region of a PBS-treated *Tg(lyz:eGFP)* larva from 1 dpi to 1.5 dpi (1 frame = 5 min). Limited expansion of lyz-GFP^+^ neutrophils is observed. Occasionally, the dividing lyz-GFP^+^ neutrophils are imaged, which are presented in 1a, 1b, 2a, 2b. The video shows the maximum projections of all planes. The time is indicated in hours and minutes. Scale bars, 20 µm.

**Video S4. The expansion and maturation of lyz-GFP^+^ neutrophils in the CHT after intravenous *E. coli* (5-10x10^3^ cfu) injection.**

This video shows time-lapse confocal fluorescence imaging of the CHT region of an *E. coli-*infected *Tg(lyz:eGFP)* larva from 1 dpi to 1.5 dpi (1 frame = 5 min). The generation of lyz-GFP^+^ neutrophils is easily observed in the regions akin to the CA. These lyz-GFP^+^ neutrophils are initially immature, as represented by their weak GFP signals. Gradually, they undergo differentiation, and the GFP signals become stronger. Compared with its control in Video S3, the dividing lyz-GFP^+^ neutrophils are frequently imaged. In total, 8 dividing lyz-GFP^+^ neutrophils are observed, which are presented in 1a, 1b, 2a, 2b……, 8a, 8b. The bacterial clots are also monitored (red signals). The video shows the maximum projections of all planes; selected frames are shown in Figure 2F. The time is indicated in hours and minutes. Scale bars, 20 µm.

**Video S5. Expansion of mpeg1-GFP^+^ macrophages in the CHT after intravenous *E. coli* (5-10x10^3^ cfu) injection.**

This video shows time-lapse confocal fluorescence imaging of the CHT region of an *E. coli-*infected *Tg(mpeg1:eGFP)* larva from 1 dpi to 1.5 dpi (1 frame = 5 min). The expansion of mpeg1-GFP^+^ macrophages is observed. These mpeg1-GFP^+^ macrophages are initially immature, as represented by their weak GFP signals. Gradually, they undergo differentiation, and the GFP signals become stronger. In addition, the phagocytosed macrophages are monitored (red signals). The video shows the maximum projections of all planes; selected frames are shown in Figure 4F. The time is indicated in hours and minutes. Scale bars, 20 µm.

**Video S6. Efficient clearance of bacteria in the *Tg(lyz:eGFP)* CHT after intravenous *E. coli* injection (5-10x10^3^ cfu).**

This video shows time-lapse confocal fluorescence imaging of the CHT region of an *E. coli-*infected *Tg(lyz:eGFP)* zebrafish larva from 6 hpi to 19 hpi (1 frame = 5 min). The circulating *E. coli* are engulfed by both macrophages (white cross star) and neutrophils (white stars); thus, free DsRed^+^ *E. coli* (white arrowheads) clearly reduce. The video shows only one confocal plane. The time is indicated in hours and minutes. Scale bars, 20 µm.

**Video S7. Inefficient phagocytosis of bacteria in the *pu.1^G242D/G242D^/Tg(lyz:eGFP)* CHT after intravenous *E. coli* (5-10x10^3^ cfu) injection.**

This video shows time-lapse confocal fluorescence imaging of the CHT region of an *E. coli-*infected *pu.1^G242D/G242D^/Tg(lyz:eGFP)* larva from 6 hpi to 19 hpi (1 frame = 5 min). Compared with its control in Video S6, the lack of macrophage causes the inefficient bacterial phagocytosis (white cross star). Large portion lyz-GFP^+^ neutrophils perform the engulfment and numerous small bacterial foci are formed (white arrowheads). Resultantly, large amounts of free DsRed^+^ *E. coli* circulate in the vessels for longer periods. The video shows only one confocal plane; selected frames are shown in Fig 6A, which include images from single z-planes of the same time-lapse confocal series. The time is indicated in hours and minutes. Scale bars, 20 µm.
